# Pyrazolyl-Ureas as Interesting Scaffold in Medicinal Chemistry

**DOI:** 10.3390/molecules25153457

**Published:** 2020-07-29

**Authors:** Chiara Brullo, Federica Rapetti, Olga Bruno

**Affiliations:** Department of Pharmacy, Section of Medicinal Chemistry, University of Genoa, Viale Benedetto XV 3, I-16132 Genova, Italy; federica.rapetti@edu.unige.it (F.R.); obruno@unige.it (O.B.)

**Keywords:** pyrazolyl-ureas, pyrazole nucleus, protein kinase inhibitors, anti-inflammatory agents, anticancer agents, anti-pathogens agents

## Abstract

The pyrazole nucleus has long been known as a privileged scaffold in the synthesis of biologically active compounds. Within the numerous pyrazole derivatives developed as potential drugs, this review is focused on molecules characterized by a urea function directly linked to the pyrazole nucleus in a different position. In the last 20 years, the interest of numerous researchers has been especially attracted by pyrazolyl-ureas showing a wide spectrum of biological activities, ranging from the antipathogenic activities (bacteria, plasmodium, toxoplasma, and others) to the anticarcinogenic activities. In particular, in the anticancer field, pyrazolyl-ureas have been shown to interact at the intracellular level on many pathways, in particular on different kinases such as Src, p38-MAPK, TrKa, and others. In addition, some of them evidenced an antiangiogenic potential that deserves to be explored. This review therefore summarizes all these biological data (from 2000 to date), including patented compounds.

## 1. Introduction

Pyrazole consists of a doubly unsaturated five-membered ring containing two nitrogen atoms (named N1 and N2) and, among heterocyclic compounds, represents one of the most important chemical scaffolds in medicinal chemistry and advanced organic materials [1].

The biological and pharmacological properties of pyrazole can be due to its particular chemical characteristics: in detail, pyrazole presents a nitrogen atom 1 (N1), also named “pyrrole-like” because its unshared electrons are conjugated with the aromatic system; and a nitrogen atom 2 (N2), named as “pyridine-like” since the unshared electrons are not compromised with resonance, similarly to pyridine systems. Due to the differences between these two nitrogen atoms, pyrazole can react with both acids and bases [2]. Another important structural characteristic of pyrazole is the prototrophic tautomerism: in fact, three tautomers are possible in unsubstituted pyrazole, while five tautomers can exist in mono-substituted pyrazoles [3]. 

Pyrazole and its derivatives exhibit a broad spectrum of pharmacological activities from antimicrobial and antitubercular to anticonvulsant, anticancer, analgesic, anti-inflammatory, antidepressant, cardiovascular, and many others [4].

Examples of the most recent pyrazole drugs are Celecoxib, Lonazolac, Tepoxalin, Deracoxib, Mepirizole, Crizotinib, Pyrazomycin, Surinabant, Rimonabant, Difenamizole, Fezolamine, Betazole, and Fomepizole, among many others. All these molecules are uncondensed variously substituted pyrazoles, but condensed pyrazole rings are also an important source of bioactive molecules [5,6].

In recent years, the urea function has led to the development of many drugs that are especially useful in anticancer therapy such as Sorafenib, Cabozatenib, and Regorafenib [7]. In the last ten years, various urea derivatives have been studied as biological modulators of different intracellular targets, confirming the importance of the urea scaffold in medicinal chemistry and in drug development. In fact, the urea NH moiety is a favorable hydrogen bond donor, while the urea oxygen atom is regarded as an excellent acceptor, giving to the function the possibility for interacting with several protein targets in different ways. In addition, due to strong intermolecular hydrogen bonding with different solvents, insertion of the urea moiety can enhance aqueous solubility.

When a urea function was inserted on the pyrazole nucleus in position 3, 4, or 5, different biologically active compounds with a broad spectrum of pharmacological properties were obtained, from antibacterial or antiparasitic to anti-inflammatory and, above all, anticancer ones [8].

This review focuses on the study and classification of new pyrazolyl-ureas (appeared in the literature over the past 15 years) and their interesting pharmacological properties. The numerous articles and patents have been classified firstly on the base of the molecules structure (in particular, the position of the urea function in the pyrazole scaffold has been used as discriminant factor), and then on the base of the most relevant bio-pharmacological activities reported by the authors.

## 2. 3-Pyrazolyl-Ureas Derivatives

### 2.1. 3-Pyrazolyl-Ureas as Human Carbonic Anhydrase Inhibitors

**Figure 1:**
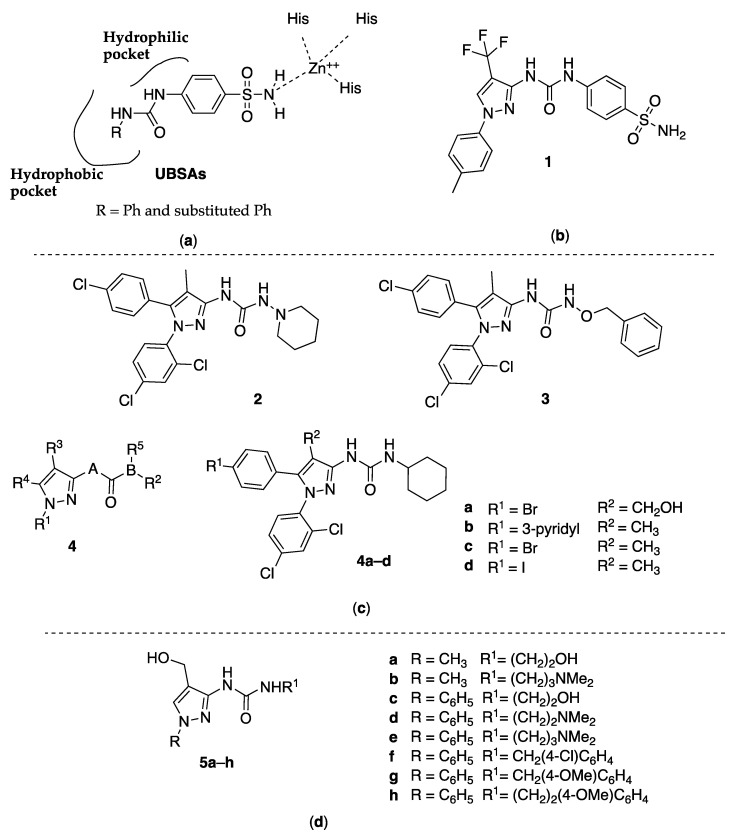
(**a**) General structure of UBSAs and their localization in the binding pocket of hCA (adapted from Sahu, 2013 [10]). 3-pyrazolyl-urea reported as: (**b**) hCA inhibitors; (**c**) CB1/CB2 receptor antagonists; (**d**) antibacterial/antifungal agents.

Metabolic transformations such as the reversible hydration of CO_2_ to HCO_3_^−^, aldehyde hydration, hydrolysis of alkyl and aryl esters, urine formation, and other similar physiological and physiopathological reactions are catalyzed by the human carbonic anhydrase II (hCA II), a metalloenzyme that uses zinc to catalyze these transformations. In detail, a tetrahedral zinc ion (Zn^2+^) coordinated with three histidine residues and one water molecule represents the catalytic domain, which is the primary target of human carbonic anhydrase inhibitors (hCAIs). Sulfonamide or sulfamate group linked to an aromatic or heteroaromatic ring seems to play an important role in binding to the Zn^2+^, as proven by the most powerful inhibitors Celecoxib, Acetazolamide, and ureido-benzenesulfonamides (UBSAs, Figure 1). 

Supuran and coworkers recently prepared, as inhibitors of the hCA II, several UBSA derivatives which, shifting the hydroxide molecule due to the sulfonamide nitrogen, bind directly to the active side, masking it [9]. They also reported very important structure–activity relationship (SAR) information about the R group orientation in the different subpockets of the active site and the capacity of linking the Zn^2+^, probably due to the flexibility of the ureido linker (Figure 1).

On this basis, Sahu and coworkers designed a new set of UBSA ligands with different substituents on the ureido group, including the 3-(1-*p*-Tolyl-4-trifluoromethyl-1*H*-pyrazol-3-yl) moiety (compound **1**, Figure 1). The crystallographic structure of hCA was used to perform a theoretical investigation of electronic structure parameters of hCA II complexed with the new molecules. In particular, the authors used their N-layered integrated molecular orbital and molecular mechanics (ONIOM) method [10]. 

The analyzed compounds were effectively able to interact with hCA II and showed calculated inhibition constants consistent with the experimental data. The study evidenced that the nature of the R moiety, substituting the second ureido nitrogen in UBSAs, controls the inhibitor potency. This is probably due to the flexibility of the ureido linker and the possibility of the orientation of the R group in different subpockets of the active site cavity. In addition, it was evident that the metal–ligand bond distances and the ligand–metal–ligand angles for **1** were in reasonable agreement with the other sulfonamide ligand, therefore indicating that **1** has a similar binding mode with other sulfonamide inhibitors. The best hCA II inhibitor was the 2-isopropyl-phenyl-substituted compound, but **1** was also revealed as a potent inhibitor of hCA II like other UBSA inhibitors, having a calculated inhibition constant of 220.93 or 224.69 nM, following the optimized geometry at the M1 or M2 level, respectively, in the docking analyses.

### 2.2. 3-Pyrazolyl-Ureas as Cannabinoid Receptor Antagonist

The cannabinoid (CB) receptors are G protein-coupled receptors which respond to cannabinoids, such as Δ-9-tetrahydrocannabinol^9^-THC), the major active constituent extracted from *Cannabis sativa*. The most important and studied are two cannabinoid receptor subtypes named CBl and CB2. Of these, CBl receptors are also known as central cannabinoid receptors because of their primary expression in the central nervous system (CNS).

In detail, CB1 receptors are mainly located in the brain (in the basal ganglia, hippocampal cell layer, cerebellum, and cerebral cortex), spinal cord, and peripheral nervous system. CB1 is also the most widely expressed G protein-coupled receptor in the brain. On the other hand, CB2 receptors are minimally expressed there; they are found mostly in the peripheral neurons, the reason for which they are named peripheral cannabinoid receptors. They block the release of neurotransmitters and were found principally in cells related to the immune system. Besides the different organ distribution, the two subtypes differ also for the aminoacidic sequence, signal transduction mechanism, and sensitivity to certain agonists and inhibitors.

Molecules acting as unselective agonists against CB1 and CB2 receptors have undesirable side effects such as drowsiness and impairment of monoamine oxidase function and of non-receptor mediated brain function. The addictive and psychotropic properties of some cannabinoids also limit their therapeutic value. On the contrary, compounds having antagonist activity could provide a pharmacological response useful to treat cannabis abuse, lipid and glucose metabolic disorders, cardiovascular disorders, psychotic disorders (stress, anxiety schizophrenia), epilepsy, migraine, vomiting, memory and cognitive disorders related to neurodegenerative diseases, and more. 

Different studies on endogenous ligands have also shown that CB1 regulated food intake and energy consumption for weight management purposes. These studies led to the development, by Sanofi-Aventis, of the first CBl receptor antagonist, Rimonabant, approved in the UK in July 2006 for the treatment of obesity and as a smoking cessation drug. Because of its serious central side effects, the research is now more interested in the discovery of new peripherally selective CBl receptor antagonists.

With this intention, Li and researchers [11] tried to find new small molecules as CBl antagonists with a 3-pyrazolyl-ureas substituted scaffold and also performed the activity test on EGFP-CB1_U20S cells expressing the CBl receptor. From these tests, the IC_50_ values of the compounds ranged from 5 μM to 10 μM, of which compounds **2** and **3** (Figure 1) were the best CB1 receptor antagonists with IC_50_ values of 5.19 and 3.61 nM, respectively.

Another patent was filled by Makriyannis and coworkers in 2006. It related to biologically active pyrazole analogs having Markus structure **4** (Figure 1), acting as antagonists for CBl and/or CB2 receptors and having selectivity for one of them [12]. Some of the inventive compounds showed high affinity for at least one of the cannabinoid receptors. The CB1 receptor binding affinities (*K*_i_) for the synthesized analogs ranged between 6 and 1844 nM, while the CB2 *K*_i_ ranged between 36.5 and 13,585 nM. Interestingly, some analogs were able to interact with the CBl receptor without affecting the peripheral (CB2) receptor to the same degree. On the contrary, other analogs were able to interact with the CB2 receptor without affecting the CBl receptor. The most interesting were the pyrazolyl-ureas **4a**–**d** (Figure 1) as shown by affinity constant (*K*_i_) and selectivity reported in Table 1.

### 2.3. 3-Pyrazolyl-Ureas as Antibacterial Agents

Among 3-ureido-substituted pyrazoles, several examples were functionalized at the ring position 4 with a methyl, ethoxycarbonyl, or carboxy group and were obtained by adding alkyl- or arylisocyanates to the respective 4-aminopyrazoles. Aiming to insert a hydroxymethyl function in position 4, Bratenko and coworkers considered this approach less appropriate, because 3-amino-4-hydroxymethylpyrazoles were unknown in the literature, and even if such compounds were available, selectivity problems would arise when adding isocyanates to the amino group. On this basis, they developed an effective method for the preparation of 1-alkyl(aryl)-3-[4-(hydroxymethyl)-1*H*-pyrazol-3-yl]urea derivatives (**5a**–**h**, Figure 1) based on the interaction of 4-(hydroxymethyl)pyrazole-3-carbonyl azides with primary aliphatic or aromatic amines under Curtius reaction condition. Then, compounds **5** were evaluated as antibacterial and antifungal agents against *Staphylococcus aureus* (ATCC 25923), *Escherichia coli* (ATCC 25922), *Bacillus subtilis* (ATCC 8236F800), and *Candida albicans* (ATCC 885-653), showing a moderate antimicrobial activity. In detail, all the compounds tested present minimum bacteriostatic/fungistatic (MIC), bactericidal (MBC), and fungicidal (MFC) concentrations of 250 μg/mL [13].

## 3. 4-Pyrazolyl-Ureas Derivatives

### 3.1. 4-Pyrazolyl-Ureas as Epoxide Hydrolase (sEH) Inhibitors

**Figure 2:**
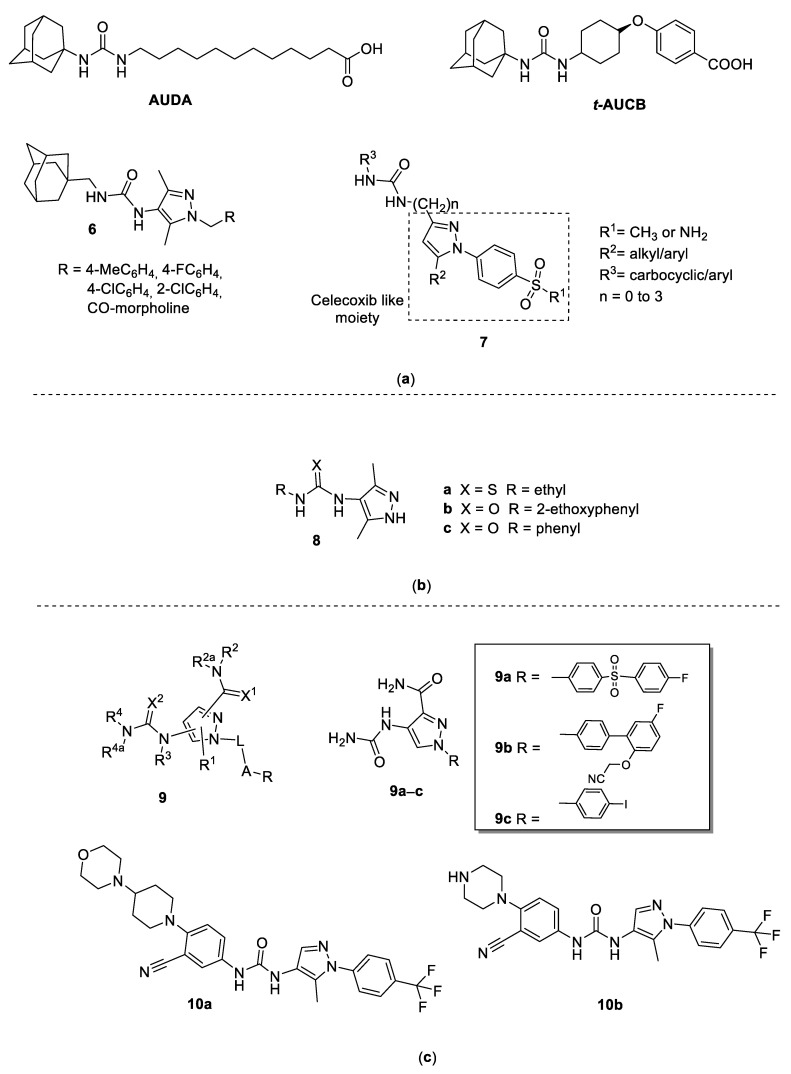
4-Pyrazolyl-ureas reported as: (**a**) sEH inhibitors; (**b**) anticonvulsant; (**c**) anti-inflammatory agents.

In mammals, epoxide hydrolases (EH) catalyze the transformation of several carcinogenic epoxides into their corresponding nontoxic diols. Soluble EH (sEH) is also involved in the metabolism of arachidonic and linoleic acid epoxides, and the diols derived from epoxy-linoleates (leukotoxins) have been associated with several inflammatory and vascular pathologies. Therefore, sEH has been suggested as a promising pharmacological target for potential anticancer and anti-inflammatory agents. sEH consists in two globular proteins in which C-terminal domains can bind the different substrates to exerts the hydrolyzing action. 1,3-dicyclohexylurea was reported in 1999 by Morisseau and co-workers as the first stable inhibitor of sEH [14] and numerous symmetrical and asymmetrical diphenyl ureas have been developed [15], giving three new generations of molecules characterized by improved potency and pharmacokinetic properties. Among them, asymmetrical ureas with a flexible side chain, such as 12-(1-adamantan-1-ylureido)-dodecanoic acid (AUDA, Figure 2), have been proposed to increase solubility [16]. In 2019, in pursuing the synthesis and the study of pharmacological properties of adamantyl-containing 1,3-disubstituted ureas, D’yachenko and coworkers [17] reported the synthesis of a series of 1,3-disubstituted ureas containing the 1,3,5-disubstituted pyrazole fragment as possible sEH inhibitors (compounds **6**, Figure 2). Authors declare an inhibitory activity ranging from 16.2 to 50.2 nmol/L and increased bioavailability, while major detailed information about structure–activity relationships is lacking.

Starting from the pyrazole structure of the well-known COX-2 selective inhibitor Celecoxib and the adamantyl urea function of the sEH inhibitor *t*-AUCB (Figure 2), Hwang and coworkers [18] designed new interesting chimera compounds in which an aryl/adamantyl-substituted urea moiety is linked to the pyrazole nucleus in position 3 directly or through an alkyl chain, as reported in general structure **7** (Figure 2). Compounds having the biarylpyrazole ring and the urea group directly connected showed poor sEH inhibitory activity. On the contrary, the addition of methylene groups (total of three) between the COX-2 and the sEH pharmacophore yielded an overall twentyfold improvement in potency against sEH, without changing the efficacy against COX-2. The most active resulted compound had R^1^ = NH_2_, R^2^ = phenyl, R^3^ = *p*-(trifluoromethyl)phenyl, and three carbon atoms in the chain, which showed IC_50_ values of 0.9 nM, 1.26 μM and >100μM against sEH, COX-2, and COX-1, respectively. These results suggested that the 1,5-diarylpyrazole group is sterically hindered and, by increasing the length of the linker between the 1,5-diarylpyrazole group and the urea group, it is possible to avoid steric repulsion. These compounds have been classified in this subchapter to simplify the reading, despite the presence of some 3-pyrazolyl-ureas in the series.

### 3.2. 4-Pyrazolyl-Ureas as Antiepileptic Drugs

The incidence of epilepsy in the worldwide population is estimated to be about 0.5–1%, 70% of whom achieve satisfactory seizure control thanks to the large number of new drugs recently approved (e.g., Lamotrigine, Gabapentin, Tiagabine, and Milacemide). However, the need for more effective and less toxic antiepileptic drugs still exists since a large number of patients are drug-resistant. Urea and thioureas derivatives [19,20] have emerged as structurally novel anticonvulsant agents. It was hypothesized that ureas and thioureas display anticonvulsant activity by interacting on the putative aryl binding site, the hydrogen bonding domain, and an auxiliary aryl or other hydrophobic binding site [21]. On these bases, urea and thiourea moieties were attached to a pyrazole ring and to an aryl or alkyl group obtaining compounds **8** (Figure 2) [22]. The anticonvulsant activity of the new compounds was determined using pentylenetetrazol-induced seizure (PTZ) and maximal electroshock seizure (MES) tests, two rodent models widely used to predict protection against generalized tonic-clonic and generalized absence seizures in humans. Thiourea derivative **8a** displayed noteworthy activity in both PTZ and MES screens, while the urea derivatives **8b** and **8c** were much more potent than the thiourea derivatives (82 and 80% protection at 25 mg/kg, respectively). In addition, **8b** was found more effective than **8c** in terms of MES, and both urea derivatives gave good protection when given intraperitoneally at 25 and 50 mg/kg. 

### 3.3. 4-Pyrazolyl-Ureas as Anti-Inflammatory Agents

A large number of compounds were patented in 2005 by Pharmacia Corporation [23] as potential drugs for inflammatory diseases, in particular as inhibitors of IKK-2, an IkB kinase involved in the inflammatory response that is the main cause of rheumatoid arthritis (RA) onset. 

The transcription factor NF-κΒ, which plays a prominent role in immune and inflammatory responses, is normally sequestered in an inactive form in the cytoplasm by a member of the IkB inhibitory proteins, and this prevents the transcription of responsive genes in the nucleus. Cell stimulation leads to the phosphorylation and degradation of IkB, thereby releasing NF-κΒ to the nucleus for activation of gene transcription. The IkB kinases (IKK-1 and IKK-2), which phosphorylate IkB and thereby initiate its degradation, represent the critical, common denominator in the activation of NF-κΒ and have been considered as useful targets for new anti-inflammatory agents.

On the basis of literature evidence that active IKK-2 inhibitors could be useful for the treatment of inflammatory diseases [24] and that pyrazolecarboxamide derivatives and other heteroaromatic carboxamide derivatives are useful IKK-2 inhibitors [25], Clare and coworkers patented compounds having the Markus structure **9** (Figure 2) [23] in which a carboxamide and ureas/thioureas function were inserted alternatively in position 3 or 4 of the pyrazole nucleus. In addition, the N1 in the pyrazole was generally aryl substituted. Among the 260 examples reported in the patent compounds, **9a**, **9b**, and **9c** (Figure 2) resulted the most active, having IC_50_ values of 0.013, 0.044, and 0.067 μM, respectively, against human IKK-2, and IC_50_ values of 0.023, 0.033, 0.089 μM, respectively, against rat IKK-2.

In 2014, Sharma and co-inventors [26] patented the preparation of compounds having different heterocyclic moieties (triazole, pyrazole, imidazole, oxazole, isoxazole, thiazole, and pyrrole) linked by a urea function to five- or six-membered hetero or heteroaryl systems. The invention claimed compounds as interleukin 17 (IL-17) inhibitors and tumor necrosis factor alpha (TNFα) inhibitors and further related to their use in the treatment of inflammatory or autoimmune diseases and other related pathologies.

Among the numerous claimed compounds, the 1-(4-trifluoromethylphenyl)-5-methyl-4-pyrazolyl-ureas **10** (Figure 2) exhibited the best IL-17 and TNFα inhibitory activity in the cytokine release assay. In particular, compounds **10a** and **10b** showed IC_50_ values ranging from 0.1 to 1 μM against both IL-17 and TNFα production.

### 3.4. 4-Pyrazolyl-Ureas as Protein Kinases Inhibitors

#### 3.4.1. CDK8 Inhibitors

Cyclin-dependent kinase 8 and cyclin C (CDK8/CycC) represent a potent kinase oncogene system involved in transcriptional activity and regarded as an attractive drug target. In particular, selective inhibitors could decrease mitogenic signals in cancer cells with reduced toxicity toward normal cells. Type I ligands are able to bind the kinase in the active conformation (DMG-in) and occupy the ATP-binding site, while type II ligands bind to the inactive conformation (DMG-out) and occupy the ATP-binding site and the allosteric site (deep pocket). By rearrangement of the DMG motif, the CDK8 changes from the inactive to the active state. The deep pocket (inaccessible in the DMG-in conformation) is the binding site for many well-known kinase ligands, such as Sorafenib and Imatinib, belonging to the type II category.

**Figure 3:**
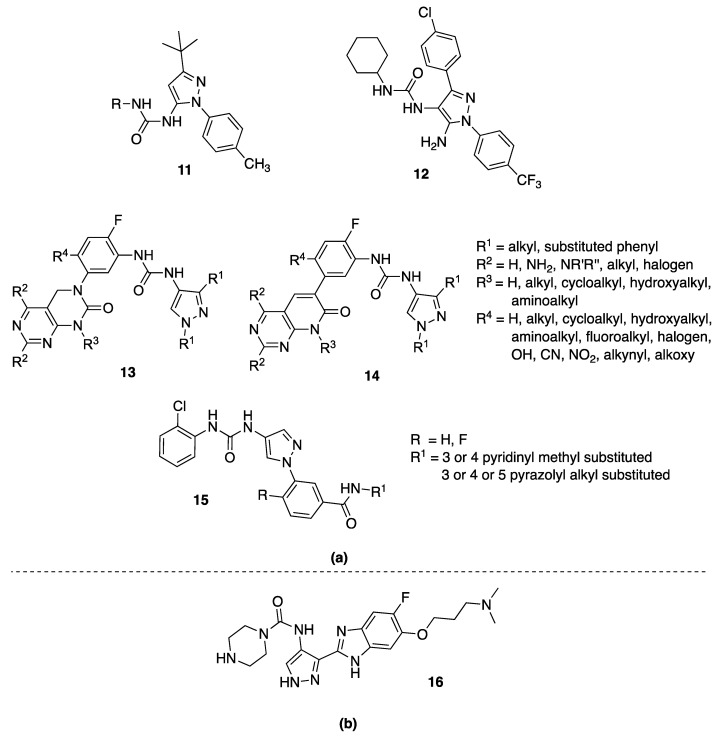
4-Pyrazolyl-ureas reported as: (**a**) protein kinases inhibitors; (**b**) anticancer agents.

The first pyrazolyl-urea series of type II CDK8 ligands was published in 2013, being actually 5-pyrazolyl-ureas variously substituted in position 4 (**11**, Figure 3) [27]. The ligands identified by Schneider et al., were found to anchor in the CDK8 deep pocket and to extend with diverse functional groups toward the hinge region and the front pocket. These variations can cause the ligands to change from fast to slow binding kinetics, resulting in an improved residence time. Major details about these inhibitors are reported in this review in Section 4 (5-pyrazolyl-ureas). 

Recently, Chen and coworkers [28] implemented a novel virtual screening protocol for drug development and applied it to the discovery of new CDK8/CycC type II ligands, starting from the pyrazolyl-ureas previously reported by Schneider et al. [27]. The authors first analyzed the thermodynamic binding of the ligands using molecular dynamics simulations and a free-energy calculation method, then extended the key binding information to assist virtual screening. Starting with the urea moiety, they carried on substructure-based searches on 187,000 compounds and, by the newly developed superposition method and single-point energy evaluation, they isolated 19 possible hits, including the 4-pyrazolyl-urea **12** (Figure 3). Finally, the free-energy calculations provided only three new compounds in which the urea moiety had the expected contacts with CDK8. Among them, the most potent drug-like compound is not a pyrazole, but a thiadiazol-urea derivative that has a *K*_d_ value of 42.5 nM, which is similar to those of the most potent pyrazolyl-urea used as a reference ligand. Interestingly, the top two compounds are different by only one atom (the phenyl urea moiety has a 4-methoxy substituent instead of a 4-ethyl one) but have a nearly threefold difference in binding affinity (*K*_i_ = 114 nM). This was accurately predicted by both energy evaluation methods. Therefore, the novel virtual screening method is accurate and useful in drug design projects.

#### 3.4.2. RAS/RAF/MEK/ERK-MAPK Inhibitors

Other interesting 4-pyrazoly-ureas, together with a large library of 5-pyrazolyl-ureas, are reported in some patents developed by Deciphera Pharmaceuticals (compounds **13** [29,30] and **14** [31,32], Figure 3). They act on different kinases especially involved in the RAS-RAF/MEK/ERK-MAPK pathways. In these compounds, the pyrazole moiety is linked by the urea function in position 4 to a phenyl, in turn substituted with a dihydropyrimido-pyrimidine scaffold (**13**, Figure 3) or the isosteric dihydropyrido-[2,3-*d*]pyrimidine (**14**, Figure 3). Biological data for these 4-pyrazolyl-urea derivatives were not given in the cited patents. Major considerations are reported in Section 4.4 focused on 5-pyrazolyl analogues.

#### 3.4.3. JNK Inhibitors

The mitogen-activated protein (MAP) kinase family member c-Jun-N-terminal kinase (JNK) has been shown to be a therapeutic target for a variety of diseases including neurodegeneration, metabolic disorders, inflammation, cardiovascular disease, and cancer. Three isoforms, namely as JNK1, JNK2, and JNK3, were identified in the JNK family. The synthesis of new JNK inhibitors has been pursued to develop efficacious therapeutics for Parkinson’s disease (PD) and other neurodegenerative diseases, such as Alzheimer’s disease (AD), Huntington’s disease (HD), amyotrophic lateral sclerosis (ALS), and multiple sclerosis (MS), but also for treatment of myocardial infarction (MI), metabolic disorders, cancer, and inflammatory diseases [33,34].

In 2015, Feng and coworkers [35] developed a large series of pyrazole derivatives aiming to obtain JNK2/JNK3 inhibitors with a high degree of selectivity in respect to the JNK1 isoform. These inhibitors were expected to afford lower toxicity risk for treatment of the conditions associated with JNK activation. Particularly, by targeting the substrate site in JNK, and potentially blocking JNK mitochondrial translocation, they hypothesized to provide an inhibition mechanism that prevents mitochondrial dysfunction and cardiomyocyte cell death.

The authors patented numerous molecules in which a substituted phenyl is always present in N1, while the pyrazole positions 3 and 5 were variously substituted. Among them, compounds of general structure **15** (Figure 3) showed the best results having, in the inhibition test, EC_50_ values <200 nM against JNK3/2, while >1000 nM against JNK1.

### 3.5. 4-Pyrazolyl-Ureas as Anticancer Agents

In 2008, Aventis Pharma patented a large series of benzimidazole-pyrazole derivatives bearing in position 4 of the pyrazole nucleus the NHCO-R moiety in which R were differently substituted alkyl- or cycloalkyl-amines [36]. Compounds were claimed as anticancer agents and were active in the cancer cells line HeLa with IC_50_ values ranging from 5 to 50 μΜ. In particular, the IC_50_ of the compound **16** (Figure 3), here reported as an example, is between 5 and 30 μΜ.

## 4. 5-Pyrazolyl-Ureas

### 4.1. 5-Pyrazolyl-Ureas as p38MAPK Inhibitors

The p38 mitogen-activated protein kinase (MAPK) is a serine–threonine kinase that includes JNK and extracellular-regulated protein kinase (ERK). It plays an important role in the regulation of a wide range of immunological responses, in particular in production and activation of inflammatory mediators (cytokines such as TNFα and IL-1β), which initiate leukocyte recruitment and activation. Consequently, it shows a crucial role in inflammation (especially in auto-immune diseases such as RA, Crohn’s disease, inflammatory bowel disease, and psoriasis), but also in cancer progression (cell proliferation, differentiation, and apoptosis). In detail, p38 is a super family divided into four different isoforms: p38α, p38β, p38γ, and p38δ. 

In the past, many p38 inhibitors with different chemical structures have been designed on the basis of their ability to compete with p38 ATP binding or allosteric site [37]. Specifically, p38MAPKα inhibitors, like many other kinase inhibitors, can be classified into two types based on their mode of action: ATP-competitive inhibitors and non-ATP-competitive, or allosteric, inhibitors, which utilize the ATP binding cleft and a hydrophobic allosteric pocket created when the activation loop adopts the inactive “Asp-Phe-Gly (DFG)-out” conformation. Because allosteric inhibitors do not compete directly with ATP or substrate, they can offer a significant kinetic advantage over ATP competitive inhibitors. In addition, because the allosteric pocket is less conserved than the ATP binding region, allosteric inhibitors usually have better kinase selectivity profiles than ATP competitive inhibitors [38].

**Figure 4:**
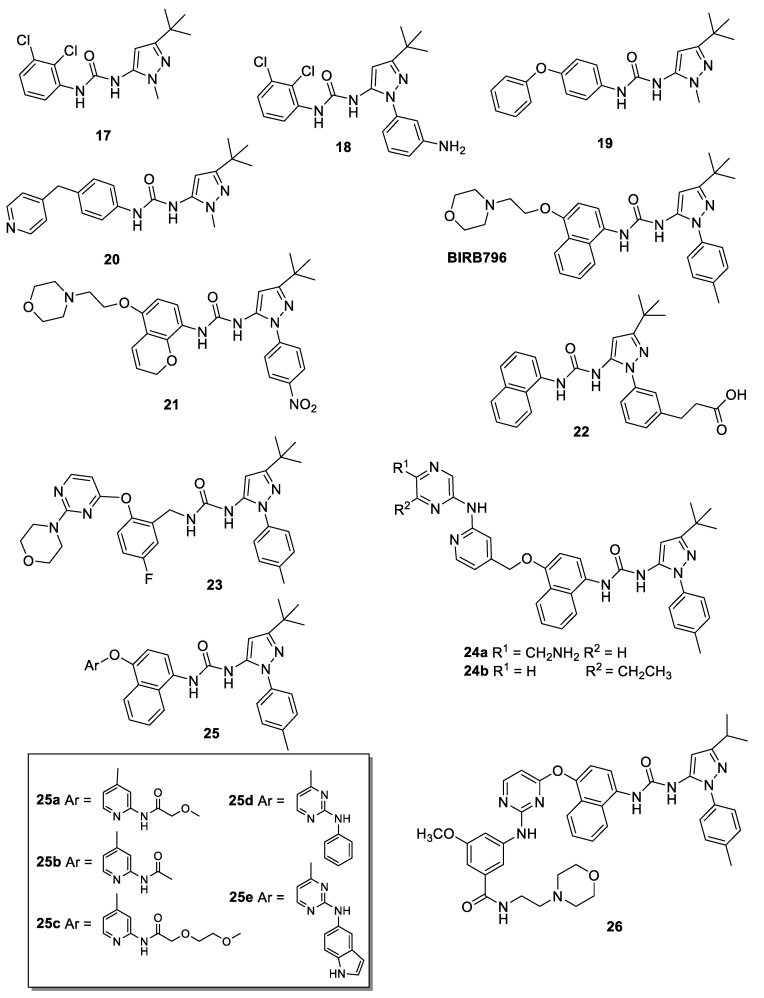
5-Pyrazolyl-ureas reported as p38 inhibitors.

The first compound discovered in 2000 by Dumas and coworkers at Bayer Pharmaceutical has been a little 5-pyrazolyl-urea which presented a *tert*-butyl substituent on C-3 and two chlorine atoms on the phenyl ring of urea moiety (compound **17**, Figure 4). It showed IC_50_ value of 53 nM on p38 enzyme and inhibited TNFα and IL-1 induced IL-6 production in SW1353 cells (human chondro-sarcoma cell line) with an IC_50_ value of 820 nM [39].

Aiming to obtain more active compounds in the cellular functional assays, the same authors synthesized a large series of 5-pyrazolyl-ureas exploring different substitution on previous lead compound. Firstly, they investigated the substitution on N1 pyrazole, leaving unchanged the urea moiety. In detail, the methyl substituent on N1 has been replaced with a more bulky and lipophilic benzene ring differently decorated. From this investigation, the authors obtained compound **18** (Figure 4), having on the N1 pyrazole a phenyl ring, carrying in turn a free amino group in meta position, that showed IC_50_ = 13 nM on p38 and 42 nM in inhibition of TNFα and IL-1 induced IL-6 production in SW1353 cells [40].

At the same time, structural changes were made on the benzene of the urea segment, removing the halogens and introducing longer and more bulky substituents, such as the phenoxy group (compound **19**, Figure 4) or the 4-methylene-pyridine one (compound **20**, Figure 4). With these structural modifications, the authors obtained p38 inhibition with IC_50_ in the nanomolar range, 270 and 42 nM, respectively, and improved in vivo activity (in acute models of cytokine release and a chronic model of arthritis), confirming that 5-pyrazolyl-urea is the best scaffold in respect to thiophene and pyrrole ones [41,42]. 

Starting from interesting results, the big pharma Boeheringer developed different 5-pyrazolyl-urea derivatives and patented 1-(3-(*tert*-butyl)-1-(*p*-tolyl)-1*H*-pyrazol-5-yl)-3-(4-(2-morpholinoethoxy)naphthalen-1-yl)urea (BIRB 796, Dorapaminod, Figure 4), one among the most active p38 MAPK inhibitors, now used as pharmacological tools in medicinal chemistry research [43,44]. 

BIRB 796 resulted the most active compound with picomolar affinity for p38 in enzymatic assay and low nanomolar inhibitory activity in cell culture [45]. In deep investigations, it showed a *K*_d_ = 0.046 nM on p38, inhibited TNFα production in THP-1 cell lipopolysaccharide (LPS)-stimulated with EC_50_ value of 18 nM and in human whole blood with EC_50_ value of 780 nM; moreover, it evidenced a good selectivity profile, inhibiting only JNK2α2 kinase with an IC_50_ value of 0.1 μM. In addition, in the LPS-stimulated TNFα synthesis mouse model, a 65% inhibition of TNFα production was observed when BIRB 796 was dosed orally at 10 mg/kg. On a model of established collagen-induced arthritis using B10.RIII mice, BIRB 796 (at 30 mg/kg os) showed a 63% inhibition of arthritis severity [45,46].

In a recent study, the authors evidenced that BIRB 796 can reduce LPS-mediated IL-8 production in THP-1 cells, but not in Raw 264.7 cells. Further analysis of signal molecules revealed that BIRB 796 sufficiently suppressed LPS-mediated phosphorylation of p38MAPK in both cell types, whereas it failed to block inhibition of kappa B (I-κB) degradation in Raw 264.7 cells. Taken together, these results suggested that the anti-inflammatory function of BIRB 796 depends on cell types [47].

In 2001, Regan and coworkers reported a new allosteric binding site for the diaryl urea class of highly potent and selective inhibitors against human p38MAP kinase. The formation of this binding site requires a large conformational change, not previously observed for any serine/threonine kinases, in the highly conserved DGF motif within the active site of the kinase. Authors demonstrated that by improving interactions in this allosteric pocket, as well as establishing binding interactions in the ATP pocket, the affinity of the inhibitors is enhanced [45].

The same authors, using X-ray crystallographic data, deeply investigated the binding kinetics of BIRB 796 to human p38α: interestingly, compound showed slow *k*_on_ and long *k*_off_ rates. In the presence of the compound, the DGF must undergo a substantial conformational change, fundamental for slow binding mode. In the binding conformation, the side chain phenyl ring of Phe169 moves about 10 Å (“DFG-out” conformation) and participates in a lipophilic interaction with the aryl portion of the inhibitor, in particular with *tert*-butyl group at C-3 of the pyrazole nucleus. This group occupies the same lipophilic pocket (Phe169 pocket) between the two lobes of the kinase which is exposed upon reorganization of the activation loop; this interaction possibly contributes to the long *k*_off_ rates.

As evidenced in the X-ray structure of BIRB 796 in p38αMAPK [45], the carbonyl of the urea moiety of BIRB796 accepts hydrogen bonds from the backbone NH of Asp168, while one of the NH forms hydrogen bonds with the Glu71 side chain. These interactions appear to be the most crucial for this class of compounds, since replacement of either NH of the urea by a methylene or an N-methyl group results in a complete loss of p38 inhibition. In addition, other important interactions are lipophilic interaction (π–CH_2_ interactions) of the pyrazole-tolyl group with the alkyl side portion of Glu71 and of the naphthyl ring system with Lys53, being that the naphthyl system is preferred as compared to simple phenyl ring; additional hydrogen bonding of the morpholine oxygen with the Met109 residue and of the ethoxy morpholine tail with ATP binding region are crucial for activity; in detail, the gauche conformation of the ethoxy linker of BIRB 796 effectively orients the morpholine group so that the morpholine oxygen can achieve a strong hydrogen bond with the NH of Met109. This modification afforded significant improvements in binding affinity, cellular activity, and in vivo reduction of TNFα production and arthritis severity. 

For all these reasons, BIRB 796 became an interesting clinical candidate for the treatment of inflammatory diseases, being subjected to 11 clinical trials (some of them in phase II investigation) now completed or terminated [48]; all these trials were focused on autoimmune diseases (RA, psoriasis, and Crohn’s disease, in particular) and also on toxicity evaluation on healthy volunteers. Unfortunately, no clinical efficacy was evidenced, but only a significant and transiently dose-dependent decrease of C-reactive protein level after one week of BIRB 796 therapy.

To obtain this compound in a more rapid and simply manner, in 2006, Bagley and co-workers reported a new synthetic approach using microwave irradiation, providing BIRB 796 rapidly in high purity [49]. 

In 2007, Kulkarni and coworkers reported a combined study of three-dimensional quantitative structure–activity relationship (3D-QSAR) and molecular docking to explore the structural insights of p38 kinase inhibitors with 5-pyrazolyl urea structure, including BIRB 796 and the major part of compounds previously reported. These 3D-QSAR studies involved comparative molecular field analysis (CoMFA) and comparative molecular similarity indices (CoMSIA). Interestingly, the authors confirmed that the hydrophobic properties are the most important for enzyme inhibition and essential structural features to design new potent analogues with enhanced activity [50].

In 2013, Schneider and co-workers, using as reference compounds BIRB 796 and other 5-pyrazolyl-ureas with very similar structure and slow binding kinetics (see compounds **11**, Figure 3), reported a structure-kinetic relationship study of CDK8)/CycC [27]. Authors evidenced that the scaffold of the 5-pyrazolyl urea compounds is anchored in the kinase deep pocket and extended with diverse functional groups toward the hinge region, causing in this way the change from fast to slow binding kinetics and resulting in an improved residence time. Differently to previous studies, the flip of the DFG motif (named “DMG” in CDK8 enzyme) to the inactive DFG-out conformation appears to have relatively little influence on the binding velocity, whereas hydrogen bonding with the kinase hinge region seems to contribute to the residence time with less impact than hydrophobic complementarities within the kinase front pocket.

More recently, according to the crystal structure of the p38MAPKα/BIRB796 complex (PDB 1VK2) Zhong and coworker synthesized a new series of compounds with the aim to preserve the activity and selectivity, and to eliminate the hepatotoxicity caused by the naphthyl ring [51,52].

They replaced the naphthalene ring of BIRB 796 with two other aromatic hydrophobic scaffolds: 2*H*-chromene and chromane. The best results have been obtained with 1-aryl-3-(2*H*-chromen-5-yl) urea derivatives, in particular with compound **21** (Figure 4), bearing on N1 pyrazole a *p*-nitro phenyl ring. Interestingly, the authors have not tested their derivatives on p38 enzyme, but only towards TNFα production in LPS-stimulated THP-1 cells. The most active compound inhibited TNFα release with an IC_50_ value of 0.033 μM, showing similar potency of BIRB 796 (IC_50_ = 0.032 μM) [53]. The same authors have previously published a short and efficient synthesis of this class of compounds, using Claisen thermal rearrangement of arylpropargyl ethers as a key step to synthesize the chromene core and obtained yield ranging from 27 to 37% [54].

Meanwhile, other compounds with very similar structure to BIRB 796 were patented by Deciphera Pharmaceuticals as p38 inhibitors, compound **22** (Figure 4) being the most representative. In detail, it bears a more hydrophilic substituent in the phenyl on N1 pyrazole and an unsubstituted naphthalene on the urea moiety, and showed an IC_50_ value of 45 nM on p38 enzyme [55,56,57]. 

In 2012 Arai and co-workers, using X-ray cocrystal studies, replaced the naphthalene group of BIRB-796 with a benzyl group to increase synthetic flexibility and to obtain more selective p38α inhibitors [58]. They reported the synthesis and pharmacological and toxicological properties of different pyrazole-benzyl urea derivatives as novel allosteric p38α inhibitors, compound **23** (Figure 4) being the most interesting. According to the docking study, the benzyl group appears to occupy the kinase specificity pocket, while the 2-morpholinopyrimidine moiety of **23** can form a hydrogen bond with Met109 in two different modes: (1) between the morpholine oxygen and the backbone NH group; and (2) between N1 of the pyrimidine ring and the same NH group. **23** exhibited highly potent p38a inhibitory activity (IC_50_ = 0.004 μM) and inhibited the production of TNFα in LPS-treated mouse in a dose-dependent manner after a single oral administration (ED_50_ = 16 mg/kg). Furthermore, a five-day repeated oral dose administration suggested that **23** has low hepatotoxicity.

Other patents by RespiVert and TopiVert Pharma in the UK were related to inhibitors of p38 MAP kinase (particularly α and γ isoforms) and Src kinase and to their use in therapy, especially in the treatment of lung inflammatory disease (such as asthma and COPD) or gastrointestinal tract pathologies (such as ulcerative colitis, irritable bowel disease, IBD, and Crohn’s disease) or uveitis.

In detail, compounds **24a** and **24b** (Figure 4) showed a long tail inserted on urea moiety and characterized by an amino-pyridinepyrazine substituent; **24a** exhibited inhibitory activities against p38α and p38γ with IC_50_ values of <0.48 nM and 16 nM, respectively [59], whereas **24b** showed IC_50_ values on p38α and p38γ of 26 and 152 nM, respectively, of 55 nM on HcK and of 199 nM on c-Src; in addition, it resulted weak inhibitor of Syk and GSK3α. Therefore, results reported in all these patents confirm the therapeutic potential of kinases inhibitors in inflammatory diseases associated with viral infections. Indeed, it was previously disclosed that compounds inhibiting both c-Src and Syk kinases were effective agents against rhinovirus replication [60] and compounds inhibiting p-Hck were effective against influenza virus replication [61] (see also Section 4.1 in this review). In 2015 Longshaw and coworkers reported different composition formulation and pharmacodynamic evaluation of **24b** [62]. In this second patent, Longoshaw and coworkers patented different formulations (for oral, topic, and aereosol administrations) of compound **24b**, which resulted in the most interesting from all previous patented compounds. Results suggested that the new formulations have similar anti-inflammatory properties to previous patented compounds but, interestingly, they showed a superior therapeutic index (data not reported).

A large library of compounds having general structure **25** (Figure 4), very similar to previous **24**, were patented as p38 inhibitors and claimed as anti-inflammatory/anti-viral agents, for the treatment of several infectious conditions, including influenza. In particular, these derivatives may be suitable for reducing viral load and/or ameliorating symptoms after infection and they may also be administered in combination with one or more other active ingredients (e.g., steroids, β-agonists and/or xanthine derivatives). Compounds **25**, very structurally related to BIRB 796, presented a naphthyl urea moiety characterized by an aryloxy embedded substituents. In detail, **25a** [63] **25b** [64] and **25c** [65] are the most representative of this library and showed an IC_50_ <5 μM against p38α enzyme.

Among this new series of derivatives, **25d** 1-(3-*tert*-butyl-1-*p*-tolyl-1*H*-pyrazol-5-yl)-3-(4-(2-(phenylamino)pyrimidin-4-yloxy)naphthalen-1-yl)urea with a more simple chemical structure was subjected to an additional patent, displaying a very different phenotype and demonstrating great inhibition versus p38MAPK (in particular α isoform, IC_50_ = 26nM) Hck (IC_50_ = 55 nM), c-Src (IC_50_ = 199 nM) without significant effect against GSK3α and Syk [66].

Finally, in another patent, the same company introduced different more embedded compounds; among them **25e** (Figure 4), which presents an additional indole group, was the most representative. All compounds demonstrated a very similar inhibitory profile in respect with the previous **25a**–**c** in kinase enzyme assays [67].

Other compounds, like **26** (Figure 4), characterized by an isopropyl substituent on C-3 and a morpholino group at the end of urea moiety, were tested for enzyme inhibition against p38 MAPKα, c-Src, and Syk, having IC_50_ values of 2 nM, <1 nM, and 3 nM, respectively. In vitro and in vivo studies, as well as pharmacokinetic analysis were then performed [68].

### 4.2. 5-Pyrazolyl-Ureas Interfering with Neutrophil Migration 

Neutrophil migration (chemotaxis) is a very complex process, which is often the origin of many autoimmune inflammatory diseases, such as RA, MS, and asthma. CXC chemokines (including IL-8, also called CXCL8) and fMLP (N-formyl-methionyl-leucyl-phenylalanine) represent the most common chemoattracting agents.

**Figure 5:**
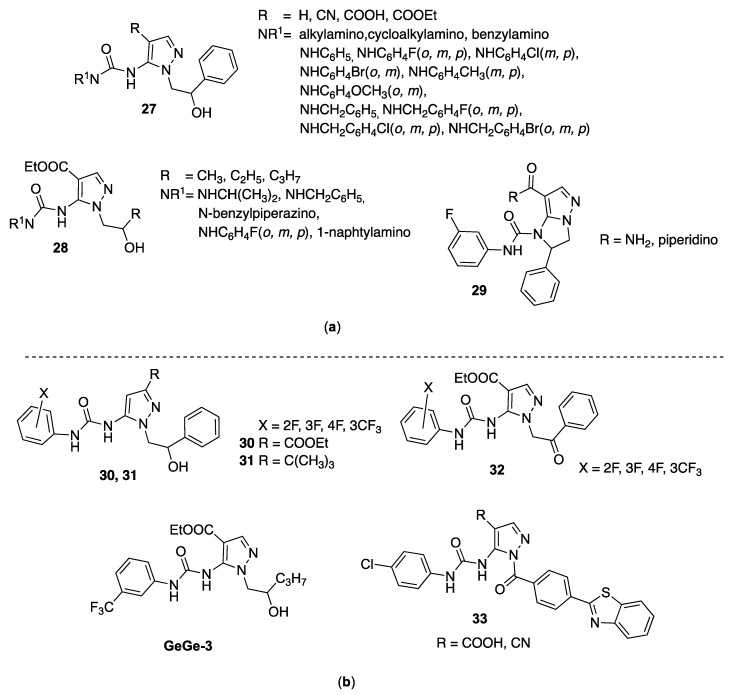
5-Pyrazolyl-ureas reported as: (**a**) chemotaxis; (**b**) antiangiogenic inhibitors.

Bruno et al. in 2007 synthesized a large series of 5-pyrazolyl-ureas (compounds **27**, Figure 5) starting from 5-amino-1-(2-hydroxy-2-phenylethyl)-1*H*-pyrazol-4-ethyl carboxylate and 5-amino-1-(2-hydroxy-2-phenylethyl)-1*H*-pyrazol-4-carbonitrile, subjected to a one-pot reaction with phosgene and proper amines or by reaction with the appropriate phenylisocyanates [69]. New *N*-pyrazolyl-*N*′-alkyl/benzyl/phenyl ureas **27** resulted very active in inhibiting IL-8 induced neutrophils chemotaxis, being the most active 3-benzyl-, 3-(4-benzylpiperazinyl)-, 3-phenyl- and 3-isopropylureido derivatives, with IC_50_ values of 10, 14, 45, and 55 nM, respectively.

Compounds **27** also resulted very selectively, as they do not inhibit fMLP-induced neutrophil migration. Regarding their mechanism of action, different biological tests were performed, being that the compounds are not able to block chemokine receptors (in detail, CXCR1 and CXCR2). The most active derivatives showed phosphorylation inhibition of protein kinase with 50–70 KD, a molecular weight, while specific binding test on different kinases having this molecular weight (Src, Fgr, Hck, Zap-70) gave negative results. Interestingly, derivatives significantly reduced the polymerized actin content and pseudopods formation, mechanisms necessary for migration. In addition, compound carrying in position 4 the carboxyethyl group and in 5 the *m*-fluoroaniline urea moiety showed a good anti-inflammatory action in vivo in a model of zymosane-induced peritonitis in mice and was able to significantly decrease the percentage of cells and granulocytes in the peritoneal cavity at a dose of 100 mg/kg os.

Subsequently, using the same synthetic method, a small library of pyrazolyl-ureas was synthesized (compounds **28**, Figure 5) carrying in position 4 the carboxyethyl group and in position 5 the urea functions more active in the previous series; hydroxyalkyl chains (less bulky than the hydroxyphenyl ethyl chain of the previous ones) were introduced in N1 position.

Many of the new compounds showed an excellent ability to inhibit both the fMLP-OMe-induced chemotaxis (a synthetic derivative of fMLP having the same chemoattracting capacities) with IC_50_ ranging from 0.19 nM to 2 μM, and the IL-8 induced chemotaxis (with IC_50_ in the picomolar range). Two of the most active compounds (bearing both a hydroxypropyl chain on N1 and an *orto*-fluoro- or *meta*-fluorophenyl on the urea moiety) have therefore been tested in vivo, as has the previous, in the zymosane-induced peritonitis inflammation model in mice. At a dose of 50 mg/kg os, they reduced the absolute and relative infiltration of granulocytes into the peritoneal cavity, confirming a possible interference in different neutrophil mechanisms at the inflammation site [70].

During subsequent deep investigations, the authors demonstrated that compounds (in particular some bearing a *meta*-fluoroanilino substituent on the urea scaffold) interfered with ERK1/2, Akt and p38MAPK phosphorylation signaling pathways [71]. Given the interesting results obtained, pyrazolyl-urea molecules by Bruno and coworkers have been patented [72].

In pursuing the investigations in this topic, the same authors synthesized a molecules series in which the urea function was inserted in an imidazo[1,2-*b*]pyrazole cycle (**29**, Figure 5), a constrained and tense form of previous 5-pyrazolyl-ureas, being this cycle not yet investigated for its biological properties [73]. These compounds also were tested in vitro for their ability to interfere with human neutrophils functions. All derivatives were inactive in inhibiting the superoxide anion production and the lysozyme release, while were very active in blocking the fMLP-OMe chemotaxis in a dose dependent manner (IC_50_ between 10 μM and 0.48 nM). In particular, compounds more active, as for previous analogues pyrazolyl-ureas, have a *meta*-fluorophenyl substituent in the urea moiety (**29**, Figure 5). Deep pharmacological investigations evidenced strong p38MAPK inhibition after activation by both fMLP-OMe or IL-8 chemoattractants, while they show different actions on PKC isoforms, suggesting that a fine tuning of the neutrophil activation occurs through differences in the activation of signaling pathways [74].

### 4.3. 5-Pyrazolyl-Ureas as Antiangiogenic Agents

Taking in account the SAR information given by previous studies, Bruno and coworkers designed and synthesized another small library of 5-pyrazolyl-ureas, combining old and new decorations, their type and position, both on the pyrazole and the urea moieties to increase the molecular diversity.

In detail, the carboxyethyl substituent was moved from position 4 to the uninvestigated position 3 (**30**, Figure 5); in another series, in position 3 was added the *t*-butyl substituent, in analogy with other well-known p38-inhibitors (**31**, Figure 5); in both series the same 2-hydroxy-2-phenylethyl chain was maintained on N1 of the pyrazole core; whereas, in order to modulate the lipophilicity of the molecules, the hydroxy-phenylethyl group was oxidized in a third series (**32**, Figure 5). As concerns the phenyl-urea moiety, different fluorinated substituents in all positions were inserted [75].

All compounds were preliminary screened to evaluate their activity on MAPK and PI3K pathways by monitoring ERK1/2, p38 and Akt phosphorylation. SAR consideration showed that specific substituents and their position on pyrazole nucleus, as well as the type of substituent on the phenyl-urea moiety, play a pivotal role in determining increase or decrease of kinases phosphorylation. Then, by a wound healing assay they were analyzed to assess their capacity of inhibiting endothelial cell migration, a function required for angiogenesis, by using human umbilical vein endothelial cells (HUVEC) stimulated by vascular endothelial growth factor (VEGF). The screening showed significant activity for compound named GeGe-3 (Figure 5). In particular, the studies confirmed that this compound might interfere with cell migration by modulating the activity of different upstream target kinases and could represent a potential angiogenesis inhibitor. Furthermore, it may be used as a tool to identify unknown mediators of endothelial migration and thereby unveiling new therapeutic targets for controlling pathological angiogenesis in diseases such as cancers.

Subsequent investigations showed that GeGe-3 inhibited HUVEC proliferation and endothelial tube formation and, interestingly, impaired inter-segmental angiogenesis during development of zebrafish embryos. In mice, GeGe-3 blocked angiogenesis and tumor growth in transplanted subcutaneous Lewis lung carcinomas. Additional screening revealed that compound could target Aurora B, Aurora C, NEK10, polo-like kinase (PLK2, PLK3), dystrophia myotonica protein kinases-1 (DMPK) and CAMK1, and the authors then speculated that GeGe-3 alters angiogenesis by targeting DMPK in tumor endothelial cells and pericytes. In conclusion, this 5-pyrazolyl-urea, blocking MAPK and PI3K pathways, strongly inhibits physiological and tumor angiogenesis [76].

In 2016, other authors reported synthesis and biological evaluation of different benzothiazoles linked to 5-pyrazolyl-urea nucleus on N1 of the pyrazole. The main objective in this study was the synthesis of new urea, indole, and triazole derivatives of 4-(1,3-benzothiazol-2-yl)-benzoyl-1*H*-pyrazole as anticancer agents able to inhibit VEGF–VEGFR2 complex formation and to suppress proliferation and survival of endothelial cells, angiogenesis and consequently cancer progression.

Some compounds (comprising 5-pyrazolyl derivatives **33**, Figure 5) showed promising cytotoxic activity against breast cancer cell line MCF-7 and inhibited human VEGF in MCF-7 cancer cell lines with comparable activity of reference compound tamoxifen [77].

### 4.4. 5-Pyrazolyl-Ureas as Anticancer Agents

**Figure 6:**
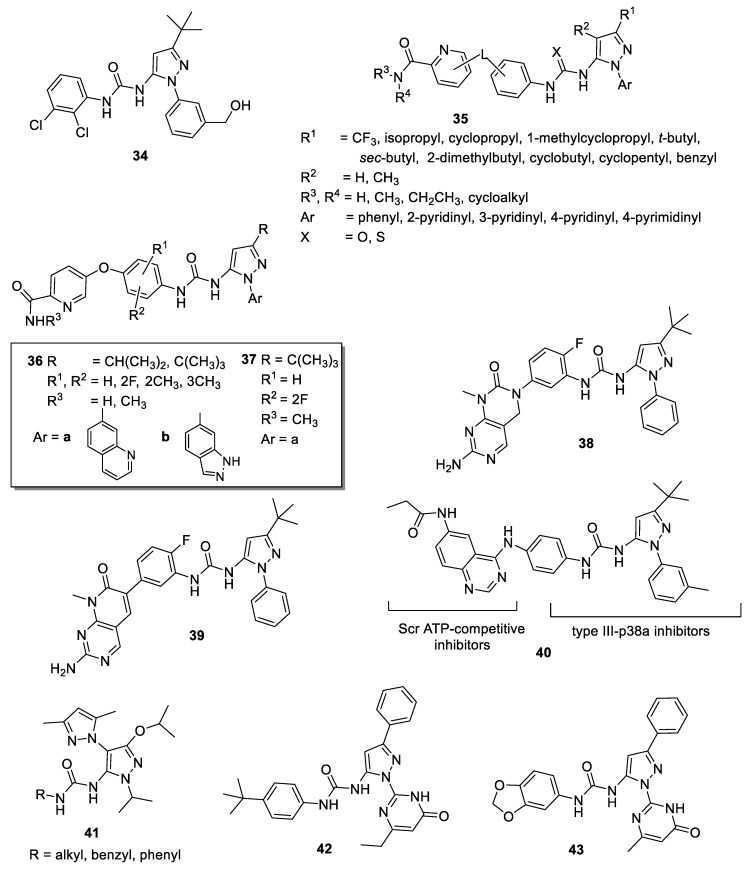
5-Pyrazolyl-ureas reported as anticancer agents.

Researchers at Deciphera Pharmaceuticals patented and synthesized other 5-pyrazolyl-ureas differently decorated on N1, C-3, and urea moiety as enzyme modulators; in detail, the major part of compounds were characterized by the 2,3-dichlorophenyl-urea moiety (**34**, Figure 6), as the molecules reported by Dumas [39,40,41,42]. They evaluated compounds not only on p38 kinase protein, but also on c-Abl, Bcr-Abl, B-Raf, VEGFR, and PDGFR, protein kinases all involved in cancer development. For some derivatives, the authors obtained good results, with IC_50_ values in the low micromolar range [78,79].

In 2007, Smith and coworkers at Bayer Pharmaceutical patented a large series of 5-pyrazolyl-ureas strictly related to BIRB 796 (compounds **35**, Figure 6) [80]. In detail, the authors introduced in position 3, in addition to the *t*-butyl group present in the BIRB 796, bulkier (benzyl, cyclobutyl, cyclopentyl), branched (cyclopropyl, *sec*-butyl) or lipophilic (CF_3_) substituents and also varied the aromatic substituent on N1. The greater difference with the lead BIRB 796 was represented by the urea function, which was elongated and characterized by two aromatic rings linked together by oxygen or sulfur atom. In particular, the second aromatic ring is a pyridine bearing in the *orto* position an amide group. The aim of this invention was to evaluate all synthesized compounds on different kinases pathways signaling (p38, but also Raf, VEGFR-2, VEGFR-3, PDGFR-beta, Flt-3), all involved in ongoing cancer, in cancer progression, and in the angiogenesis process. Furthermore, inhibition of Bcr-Abl wild type, an aberrant tyrosine kinase responsible for the onset of chronic myeloid leukemia (CML), and of Bcr-Abl T315I kinase, responsible of imatinib-resistant CML, were evaluated. Some derivatives evidenced antiproliferative properties (IC_50_ < 10 μΜ) in one or more cell lines such as CAKI-1, MKN45, HCC2998, K562, H441, K812, MEG01, SUP15, HCT116, Ba/F3-Abl (wt) and Ba/F3-Abl(T315l mutant). In detail, a compound (molecular formula not showed) exhibited interesting IC_50_ values on: CAKI-1 (5.2 μΜ), MKN45 (2.6 μΜ), HCC2998 (4.7 μΜ), K562 (<1 μΜ), H441 (4.4 μΜ), Ba/F3-Abl(wt) (<1 μΜ), and Ba/F3-Abl(T315l) (<1 μΜ).

In other patents, the same authors at Deciphera Pharmaceuticals focused on compounds with possible action on c-Abl, c-Kit, B-Raf, c-Met, Flt-3, VEGF, PDGF and HER family and potentially useful in the treatment of myeloproliferative and other similar diseases. In general, in this new large library (very similar to them patented by Smith [80]) on the N1 pyrazole was introduced a hetero-aromatic bicyclic substituent; the urea moiety was characterized by a phenyl group further substituted with a pyridine nucleus, thus giving a very long and bulky structure (**36**, Figure 6). The best biological results have been obtained for compound **37** (Figure 6) which showed an IC_50_ value of 0.8 nM for Abl and of 6 nM for T315I mutant Abl kinase. In cellular assay **37** was active on BaF3 cells expressing oncogenic Bcr-Abl kinase (IC_50_ = 6 nM), T315I BaF3 cells (IC_50_ = 8 nM), Y253F BaF3 cells (IC_50_ = 26 nM), E255K BaF3 cells (IC_50_ = 83 nM) and M351T BaF3 cells (IC_50_ = 11 nM). These five forms of Bcr-Abl kinase are oncogenic, causative of human chronic myelogenous leukemia and resistant to Gleevec^®^ [81,82]. 

Other patents focused on similar compounds bearing on the urea moiety a dihydropyrimido[4,5-*d*]pyrimidine scaffold. Compounds were claimed as potential drugs for cancer, in particular breast cancer, but also for anti-inflammatory pathologies as RA, retinopathies, psoriasis and others. In these patents, the authors reported the synthesis of new urea derivatives, inserting not only pyrazole, but also imidazole, thiadiazol and isoxazole nucleus. Interesting results have been obtained for 5-pyrazolyl-ureas (as compounds **38**, Figure 6), but also 4-pyrazolyl-urea derivatives were reported (see compounds **13**, Figure 3 [29,30], Section 3.4.2 of this review). Compounds were tested as B-Raf and C-Raf inhibitors and also on A-375 cancer cells to verify their antiproliferative activity. In general, all compounds exhibited >50% inhibition of proliferation at 1–10 μM concentration [29,30].

The same authors patented very similar compounds, isosters of previous, presenting dihydropyrido[2,3-*d*]pyrimidine nucleus instead of dihydropyrimido-pyrimidino one (**39**, Figure 6). In this larger library, the authors newly introduced the urea function on different heterocycles, such as pyrazole, imidazole, oxazole and others. As in the previous patents, also 4-pyrazolyl-ureas were reported (see compounds **14**, Figure 3 [31,32], Section 3.4.2 of this review). Compounds were claimed as useful for chronic myelogenous leukemia, acute lymphocytic leukemia, gastrointestinal stromal tumors, hyper-eosinophilic syndrome, glioblastoma and other solid tumors caused by a mutation in the RAS/RAF/MEK/ERK-MAP kinase pathway, but also for several autoimmune diseases. The invention included the compounds evaluation on Raf (isoforms A, B and C) and other kinases involved in the RAS/RAF/MEK/ERK-MAP kinase pathway. In general, compounds had >50% inhibition activity at 0.2–2 μM concentration against B-Raf and C-Raf and >50% inhibition activity at 1–10 μM concentration against A375 cancer cell line [31,32].

In 2009, Getlik and Rauh [83] designed and synthesized new molecules able to stabilize an enzymatically inactive conformation of the c-Src, a well-known kinase overexpressed or up-regulated in several solid tumors. Compounds were also active on a particular mutation (T338M) at the gatekeeper residue resistant to Dasatinib. Moreover, they seem to be more selective than ATP-competitive inhibitors, as for Scr allosteric inhibitors (type III inhibitors) that bind to sites lying outside the highly conserved ATP pocket, and may circumvent some mechanisms of drug resistance. The authors designed new type II hybrid inhibitors, starting from the analysis of crystal structures of c-Src in complex with different type III pyrazolyl-ureas (previously reported as allosteric inhibitors of p38 and resulted weakly Src inhibitors) and with type I quinazoline-based inhibitors. SAR of the new chimera derivatives confirmed that the geometry is fundamental to direct the binding to c-Src or p38 and to permit the circumvention of larger gatekeeper residue T338M. The most active compound **40** (Figure 6) inhibited wild type c-Src and T338M Src mutant with IC_50_ values of 0.014 μM and 0.023 μM respectively and in prostate carcinoma cells disrupted Fak (focal adhesions Kinase), a non-receptor tyrosine kinase involved in cell cycle progression, cell survival, and cell migration [83]. 

More recently, other authors synthesized a series of novel bis-pyrazoles with amide, sulfonamide and urea functions. In particular, compounds presented two different pyrazoles, linked together by N1 bond, one of which is 3,5-dimethyl substituted, whereas the other one has in 5 position amide, sulfonamide or urea moieties [84]. Compounds were evaluated for their in vitro cytotoxicity against Panc-1 (human pancreatic adenocarcinoma), H460 cell lines (human non-small cell lung carcinoma) by a WST-1 cytotoxicity assay. Among them the 5-pyrazolyl-ureas (compounds **41**, Figure 6) were less active than the analogue amide and sulfonamide derivatives.

Other 5-pyrazolyl-ureas were more recently synthesized as SUMO E1 inhibitors. Sumoylation is a cellular mechanism involved in the reversible modification of target proteins with a small ubiquitin-like modifier (SUMO) protein and it showed fundamental role in important cellular functions as DNA replication and repair, nuclear transport, signal transduction and proliferation. Among the sumoylation proteins, higher expression of SUMO E1 has been observed in several cancers [85].

In 2016, Kumar and Zhao studied some thiazole and pyrazole compounds, found to have moderate SUMO E1 inhibitory activity, as starting points for the development of highly potent lead compounds against cancer [86]. In detail, by using molecular docking approach, 3D shape and electrostatic potential investigation, the authors identified some 5-pyrazolyl-ureas able to block SUMO E1 with IC_50_ values in the low micromolar range, being compounds **42** and **43** (Figure 6) the most active (IC_50_ values of 13.8 and 30.4 μM, respectively). These compounds, bearing on N1 pyrazole an oxo-pyrimidinyl ring substituent, resulted more active than derivatives with methyl, cyclopentyl, aryl and aryl halide substitutions. Molecular docking evaluation revealed that **42** and **43** both occupy the SUMO E1 ATP binding pocket with the pyrazole moiety nicely overlapping with the ATP sugar ring, whereas the oxo-pyrimidine moieties overlap with the ATP adenine ring and make hydrophobic contacts with Leu49, Ile96 and Leu116 of the enzyme. However, the aryl-urea group was predicted to extend towards the opening of ATP binding pocket near Asn118, Ile343, Trp344 and Asp345. This interesting class of inhibitors presented broad scope for chemical optimization and could be used as starting points for development of more potent SUMO E1 inhibitors with therapeutic potential.

### 4.5. 5-Pyrazolyl-Ureas as TrKA Inhibitors

Tropomyosin receptor kinase (Trk) belongs to the receptor tyrosine kinase (RTK) family, which is involved in the development and maintenance of the nervous system. TrkA is a receptor for nerve growth factor (NGF) that mediates neuronal differentiation and programmed cell death prevention [87]. NGF/TrkA pathway plays a central role in the physiological function of chronic pain. TrkA activation triggers intracellular signaling cascades that increase the sensitivity of nociceptors, thus leading to chronic sensitization and pain. Consequently, NGF/TrkA pathway selective inhibition is a potential target for chronic pain treatment. 

**Figure 7:**
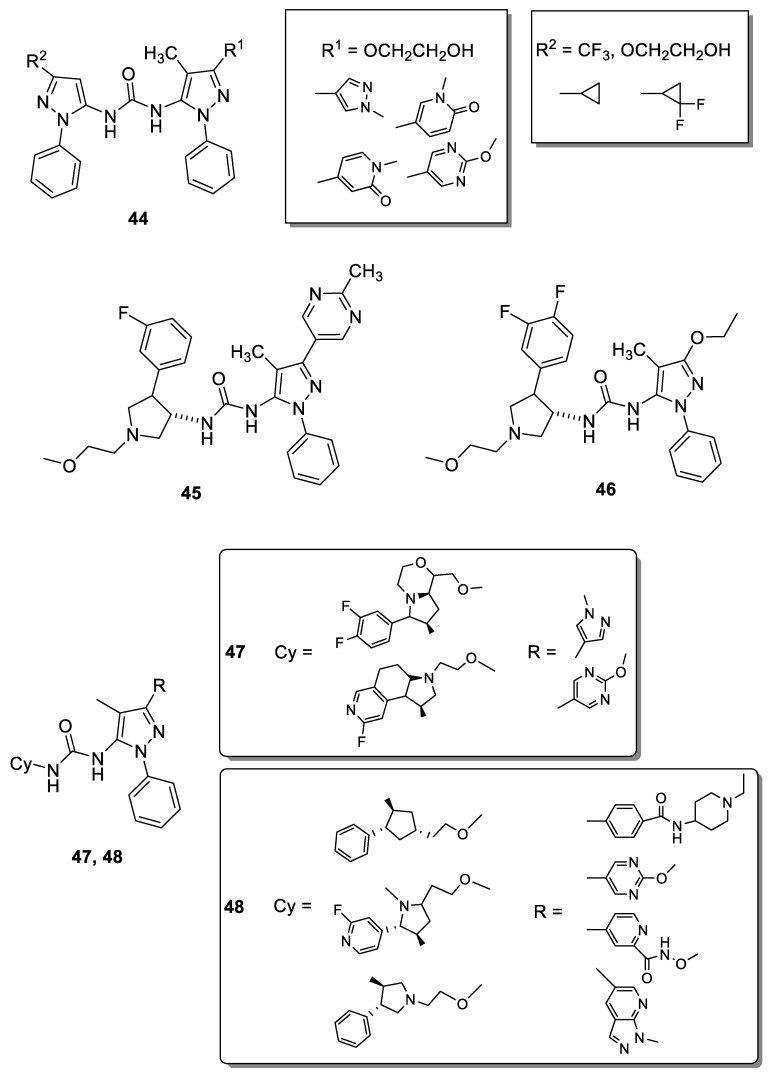
5-Pyrazolyl-ureas reported as TrKA inhibitors.

In 2014, researchers at Array Biopharma Inc. developed and patented different 5-pyrazolyl-ureas able to inhibit TrkA and claimed it as useful for the treatment of pain, cancer, inflammation, Sjogren’s syndrome, endometriosis, diabetic peripheral neuropathy, prostatitis, pelvic pain syndrome, neurodegenerative and infectious diseases, and others. Patented compounds have two pyrazole rings linked together by urea, thiourea or guanidine function, always present in position 5 of the pyrazole ring. More interesting compounds are reported in Figure 7 (compounds **44**); they showed IC_50_ values <100 nM in binding test on TrkA and evidenced also a good selectivity profile, being 1000-fold more potent against TrkA than p38α and less active versus a large panel of different kinases [88].

The same company in 2015 patented other pyrazole derivatives carrying on N1 a phenyl, on C-3 bulky aromatic groups and, in position 5, a urea, thiourea or guanidine moiety. From this large library the most interesting compound was 1-((3*S*)-4-(3-fluorophenyl)-1-(2-methoxyethyl)pyrrolidin-3-yl)-3-(4-methyl-3-(2-methylpyrimidin-5-yl)-1-phenyl-1*H*-pyrazol-5-yl)urea (**45**, Figure 7), which showed IC_50_ value of 1.1 nM in binding assay and of 1.9 nM in CHO-Kl cells transfected with human wild type TrkA. One particularly advantageous property of **45** was its predicted human clearance (which is substantially lower than other patented compounds) and its very good PK properties, such as microsomal clearance, determined in different mammalians (rat, dog and monkey). Another important aspect of **45** was its selectivity, being inactive versus hERG potassium channels. In the KinaseProfiler™ at 10 μΜ concentration **45** showed remarkable and unexpected selectivity and, interestingly, it exerted significantly different potency against TrkA versus TrkB/C. The ability of **45** to selectively inhibit the Trk pathway could indicate that it is essentially free of side-effects, which are generally related to off-target kinases inhibition, therefore representing a safer approach to treat pain, inflammation, cancer and skin diseases [89].

Recently, **45** has been subjected to a deep study by Subramanian and coworkers. The aim of this investigation was, through an initial in silico study, to decipher the allosteric binding mechanism of TrKA inhibitors, including **45**. In detail, the authors provide the basic framework for considering and selecting the appropriate in vitro screening assays or platforms to interrogate selective conformational states and explore unconventional binding pockets and interactions to achieve the desired target specificity. Extensive and exhaustive array of in vitro biochemical and biophysical tools and techniques to characterize and confirm the cryptic allosteric binding pocket and docking mode of the small hTrkA inhibitors were reported. Specifically, assays were designed and implemented to lock the kinase in a predominantly active or inactive conformation and the effect of the kinase inhibitor were probed to understand the hTrkA binding and hTrkB selectivity. The current outcome suggests that inhibitors with a fast association rate take advantage of the inactive protein conformation and lock the kinase state by also exhibiting a slow off-rate. This in turn shifts the inactive/active state protein conformational equilibrium cycle, affecting the subsequent downstream signaling. On the basis of this investigations, the authors suggested for **45** a preferential binding to the inactive enzyme conformation [90].

Similar conclusions were reported for another compound patented by Allen (**46**, Figure 6) recently investigated by other authors [91]. In detail, **46** differs from **45** for the presence of an additional fluorine atom on phenyl of pyrrolidine ring and for the substituent in position 3 of the pyrazole nucleus. To elucidate their efficiency and selectivity among the Trks, Furuya and coworkers determined the X-ray crystal structure of **46** with TrkA, including the kinase domain and Juxtamembrane (JM) region. The results revealed a novel allosteric binding mode of **46** that interacts with the JM region via hydrogen bond, van der Waals interactions, and CH-π interactions at Leu486, His489, and Ile490. In detail, Leu486 interacts with the methyl and ethoxy groups on the pyrazole moiety via van der Waals interactions; His489 interacts with the pyrazole ring via π–π interactions and with the ethoxy group on the pyrazole ring via CH-π interactions; Ile490 forms a hydrogen bond with the urea moiety and interacts with the difluorobenzene group via CH-π interactions. These three residues are not conserved in TrkB and TrkC and, consequently, should play key role for high binding affinity and selectivity. In addition, the role of the JM region in the interaction with **46** has been evaluated by a peptide-based tyrosine kinase assay using two types of TrkA kinase domains (with or without the JM region, JM+ or JM−, respectively). In conclusion, the authors classified **46** as a type III inhibitor (allosteric inhibitor) and clearly demonstrated that one of the most effective strategies for producing highly selective Trk inhibitors is the introduction on the pyrazole of a substituent able to interact with the JM region. 

In 2019, Shionogi in Japan patented two very similar classes of 5-pyrazolyl-ureas as TrkA inhibitors claimed as useful for treat osteoarthritis and RA, interstitial cystitis, chronic pancreatitis, prostatitis, and different type of pain such as chronic back pain, nociceptive, neuropathic, postoperative, pelvic, cancer pain, etc. In Figure 7 are reported **47** and **48** as representative compounds. In detail, in the first patent the most active derivatives presented a more embedded pyrrolidine nucleus on the urea moiety, resulting in a more complex structure with respect to compounds patented by Allen (**45**, Figure 7) [89]. The compounds of the present invention have not only a great TrkA inhibitory effect (IC_50_ values < 10 nM), but also a good pharmacokinetic profile, including high bioavailability and moderate clearance, high metabolic stability, high solubility, weak CYP enzyme inhibition, no mutagenic and cardiovascular action. In addition, most of synthesized compounds showed high selectivity of the TrkA receptor [92].

In compounds **48** (Figure 7), the same authors introduced in some cases a bulkier heterocyclic substituent on C-3 and a more simply pyrrolidine or cyclopentane nucleus on the urea moiety. For these derivatives also, interesting inhibitory activity on TrKA (comparable to that of compounds **47**) were obtained [93].

### 4.6. 5-Pyrazolyl-Ureas as Antimalarial Agents

#### 4.6.1. Antimalarial Agents acting as Protein-Protein Interaction Inhibitors

A critical feature of host cell invasion by apicomplexan parasites is the interaction between the carboxy terminal tail of myosin A (MyoA) and the myosin tail interacting protein (MTIP). Parasites can invade the host by a process dependent on actin and myosin machinery that is localized at the parasite’s inner membrane complex. This actin-myosin machinery is not present in human host; therefore, it is considered as an attractive drug target for parasite infectious treatment.

Among the apicomplexan parasites that use MTIP-MyoA machinery there is *Plasmodium (P.) falciparum*, the agent responsible for malaria, a devastating parasitic disease that causes more than 3 million deaths every year worldwide. The efforts to control the disease is greatly impeded by the widespread resistance that quickly emerges against the majority of drugs developed in the past and also against the most recent alternative ones. 

A fruitful strategy for overcoming microbial drug resistance is to devise new molecular scaffolds that are unrelated to current drugs and able to inhibit novel pathways exclusive for the parasite over the human host. The protein-protein interaction (PPI) between myosin motor components in apicomplexan parasites is the key to the survival of the parasite.

On this basis, Kortagere and co-workers [94,95], have designed small molecule inhibitors against this PPI by using the cocrystal structure of the *Plasmodium knowlesi* MTIP and the MyoA tail peptide. MTIP is composed of three identical subunits while the MyoA protein consists of 13 amino acids forming a hydrophobic alpha helix that occupy the binding site in the subunit C of MTIP. Using this structural information small molecules were designed aiming to competitively inhibit MyoA and disrupt the MTIP-MyoA complex.

**Figure 8:**
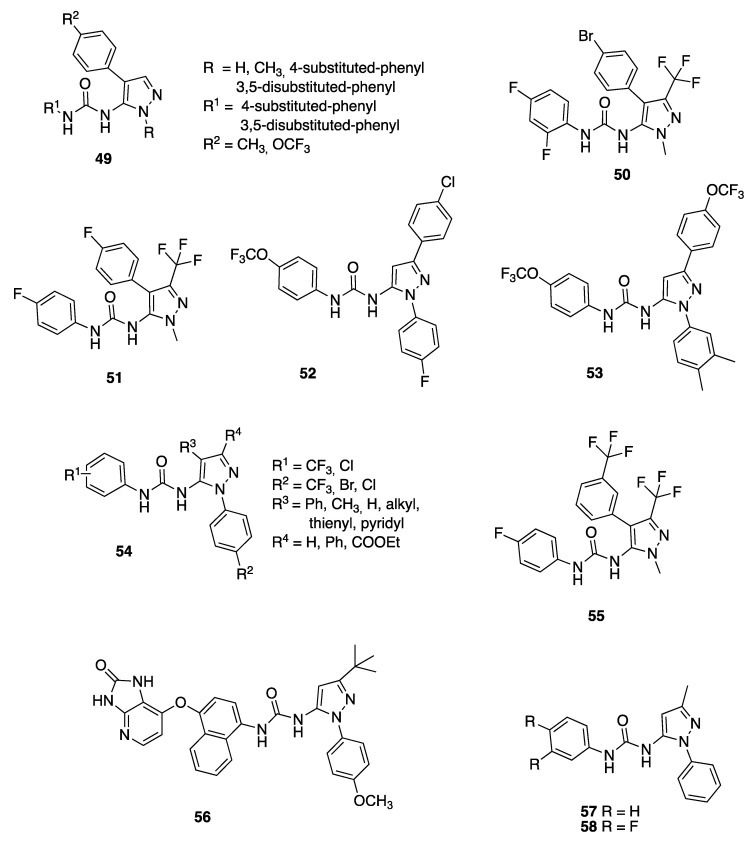
5-Pyrazolyl-ureas reported as antiparasitic agents and as potassium channel activators.

The hybrid structure-based virtual screening approach (HSB) [96] that integrates ligand-based and structure-based drug design was then applied to screen two series of small molecules. They successfully isolated eight compounds, having general structure **49** (Figure 8), belonging to the unique pyrazole-urea class, that inhibited the growth of the malarial parasite and altered the gliding motility of rodent malaria sporozoites with EC_50_ values <400 nM. Interestingly, the compounds appeared to act, at several stages of the parasite’s life cycle, blocking growth and development. The pyrazole-ureas identified in this study could be effective antimalarial agents because they competitively inhibit the key PPI between MTIP and MyoA responsible for the gliding motility and the invasive features of the malarial parasite [97].

SAR of those compounds showed that hydrophilic groups (such hydroxyl) at the *para* position on the phenyl ring in position 4 of the pyrazole ring reduced the affinity by about 30-fold. Similarly, at position R^1^, removing fluorine atoms from the *ortho* and *para* positions led to a minor improvement in the EC_50_ values. Major modification to the structure by shifting the urea linker to the C-4 carbon on the pyrazole ring yielded a compound with an EC_50_ value of about 13 μM.

#### 4.6.2. Antimalarial Agents Acting as PfATP4 Inhibitors

One of the first novel targets for antimalarial agents is the protein *Pf*ATP4, a *P. falciparum* p-type cation ATPase. P-type ATPases are important druggable targets in humans with several clinically relevant inhibitors [98,99]. *Pf*ATP4 belongs to a subfamily of these proteins (family IID) that extrude monovalent cations (sodium, lithium, and potassium). All cells must maintain low cytosolic Na+ concentration to survive and *Pf*ATP4 actively extrudes Na+ from the parasite to maintain low-[Na+]/high-[K+] in the host cell cytosol upon infection. Their presence is limited to lower eukaryotes (fungi, protozoan, and bryophytes) making them an attractive drug target for antiparasitic drugs [100,101]. *Pf*ATP4 is found in the *Plasmodium* plasma membrane and has been suggested to be the target for multiple potential antimalarial candidates, firstly for the spiroindolones, a novel antimalarial chemical class. Compound, KAE609, which is now commercially called Cipargamin, was shown to induce faster parasite clearance times than Artemisinin and was shown to be active against artemisinin resistant parasites [102].

In order to discover new molecular scaffolds acting as antimalarial, a high throughput cellular screen against *P. falciparum* asexual blood stages was performed by Flannery and coworkers on library compounds. Among them, compound **50** (Figure 8) (also known as GNF-Pf4492) showed an IC_50_ value of 184.1 nM against asexual stages of the multidrug resistant *P. falciparum* strain and demonstrated no cytotoxicity against the human hepatoma cell line Huh7 (>30 μM).

Compound **50**, wrongly defined as “aminopyrazole”, belong to the pyrazolyl-ureas already patented by Kortagere’s team [94,95]. GNF-Pf4492 blocked parasite transmission to mosquitoes and disrupted intracellular sodium homeostasis, similarly to the spiroindolones.

Flannery and co-workers applied a chemical genomic approach [103] to deeply investigate the mechanism of action of these novel antimalarial agents. Whole genome sequencing of three resistant lines showed that each had acquired independent mutations in a P-type cation-transporter ATPase, *Pf*ATP4. The authors showed that the phenotypes of parasites treated with a spiroindolone and the pyrazolyl-urea **50** are similar. They further identified a third chemotype that interacts with *Pf*ATP4. Convergence on this target by multiple chemophores highlights the critical function of this protein in the parasite [104]. 

As reported before, those pyrazole series was reported as disrupting the PPI between the *P. falciparum* MyoA and MTIP [97]. Anyway, Flannery and co-workers contested the Kortagere hypothesis about the mechanism of action, because GNF-Pf4492 was shown by Flannery as active throughout the asexual life cycle and not just during cell invasion or gliding motility, which requires the MTIP-myosin A interaction.

In our opinion, the mechanism of action of this interesting new class of antimalarial agents deserve further investigation, also to clarify whether they are not, by chance, multitargeted compounds, which would further increase their application interest.

#### 4.6.3. Antimalarial Agents Acting as *P. falciparum* Prolyl-tRNA-synthetase Inhibitors

*P. falciparum* prolyl-tRNA-synthetase (*Pf*ProRS) is a promising new target that is chemically and genetically validated by halofuginone, a synthetic derivative of the natural product febrifugine which has been known for more than 2000 years to possess antimalarial activity (IC_50_ = 275 nM). However, halofuginone is also a potent inhibitor of *Homo sapiens* prolyl-tRNA-synthetase (*Hs*ProRS) (IC_50_ = 2 μM) which prevents its use in human treatment. The overlapping of the three-dimensional structure of *Pf*ProRS and *Hs*ProRS showed that the active sites, normally occupied by proline, are identical in both enzymes. Halofuginone competes with proline in binding to the active site, making difficult to achieve specificity.

Therefore, Hewitt and coworkers [105] applied a high throughput screen (HTS) method to about 40,000 different compounds belonging to five different libraries and identified compound **51** (TCMDC-124506) (Figure 8) and Glyburide, which are very different compounds but sharing the urea function, as *Pf*ProRS inhibitors. They showed IC_50_ values against *Pf*ProRS at 34 μM and 74 μM, respectively, but <40% inhibitory activity for *Hs*ProRS up to the limit of solubility (1 mM). X-ray crystallographic structures demonstrated that these inhibitors bind outside the active site in an area of the enzyme not previously displayed as a binding pocket.

Moreover, the binding of both glyburide and TCMDC-124506 into the novel binding site significantly distorts the ATP binding site. Further analyses clearly indicated that they act as competitive allosteric inhibitors showing more than 100-fold specificity for *Pf*ProRS compared to *Hs*ProRS, demonstrating these class of compounds could overcome the toxicity of halofuginone and its analogs related to *Hs*ProRS inhibition. A large series of new variants to **51** (Figure 8) was then synthesized, guided by the cocrystal structure with *Pf*ProRS, to find more potent inhibitors while retaining selectivity against *Plasmodium* [105]. Despite the potency of those inhibitors was not greatly increased in respect with the hit (IC_50_ values ranging from 3 to 20 μM against *Pf*ProRS), interesting SAR information have been provided and opened the way for the development of new ligands as possible antimalarial agents.

In detail:

- in N1 is needed the methyl group; NH, or polar substituents are not tolerated;
- the trifluoromethyl substituent in position 3 of the pyrazole acts as vector to the solvent and its removal is not tolerated, while more polar groups (such an example 2-ethyl-morpholine) causes a potency decrease;
- removal of the *p*-fluorophenyl moiety in position 4 of the pyrazole led to inactive compounds, but substitution in the phenyl ring could be changed without affecting the potency.

Finally, the crystal structure showed that the urea moiety forms multiple H-bonds with the protein, and the aromatic substituents fit into a hydrophobic pocket. Removal of the urea led to inactive compound. The *o,p*-difluoro-substituted phenyl urea derivatives retained activity and the *m*-fluoro substitution was tolerated, whereas any other modification around this moiety is detrimental for *Pf*ProRS activity. 

### 4.7. 5-Pyrazolyl-Ureas as Anti-Toxoplasma Agents

*Toxoplasma (T.) gondii* is a ubiquitous opportunistic pathogen that infects individuals worldwide, causing severe congenital neurological and ocular disease in humans. Toxoplasmosis is a great risk for patients who are undergoing treatment for cancer and auto-immune diseases, or recipients of tissue or organ transplants. Toxoplasmosis can also lead to morbidity and death in HIV/AIDS patients. No vaccine to protect humans is available, and hypersensitivity and toxicity limit the use of the few available medicines. Therefore, safer and more effective drugs to treat toxoplasmosis are urgently needed.

Starting from their study on MTIP-MyoA that provided new antimalarial agents (see Section 4.6.1) and supported by the hypothesis that the MTIP-MyoA complex is essential also in the in *T. gondii* survival, Kortagere and co-workers constructed a hypothetical structure model of this complex and used the HSB method to screen for small molecule drugs against toxoplasmosis [106]. In the absence of the X-ray crystal structure for the *T. gondii* MTIP-MyoA complex, homology modeling techniques were employed to build a structural model of the *T. gondii* MTIP-MyoA_tail complex using the published X-ray crystal structure of *Plasmodium* MTIP-MyoA tail as the template. The MTIP-MyoA tail binding region was explored to identify the specific points of interaction between corresponding chemical groups in the MTIP pocket and the MyoA helix. The in silico screen of a chemical library containing over three million commercially available compounds made the identification of 150 hits that were submitted to the HBS analyses. Further screening through ADMET filter gave the identification of the 25 best ranking drug-like compounds and led to the discovery of the pyrazolyl-urea **52** (Figure 8) as a lead compound, with an IC_50_ value of 500 nM. In vitro assays have revealed that **52** markedly limits intracellular growth of *T. gondii* tachyzoites but has no effect on host cell human foreskin fibroblasts (HFF) at concentration more than one log greater than the concentration that inhibits the parasites. SAR analyses made information for substituents in the pyrazolyl-urea core to identify a second generation of **52** like compounds. Finally, six new molecules were synthesized and tested on *T. gondii* tachyzoites. Compound **53** (Figure 8), already reported as active against *P. falciparum* [97], performed significantly better than the parent compound in the *T. gondii* inhibition assay. Interestingly, **52** and **53** have nearly similar IC_50_ values against *T. gondii* and *P. falciparum* and may be working through similar mechanism that warrant consideration as broad-spectrum inhibitors of apicomplexan parasites.

### 4.8. 5-Pyrazolyl-Ureas as Antibacterial Agents

The emergence of resistant strains of pathogenic bacteria make mandatory the development of new antibacterial compounds. In an effort to discover new leads, Kane and coworkers [107] screened mixtures of compounds belonging to their chemical libraries versus several clinically relevant Gram-positive and Gram-negative organisms. Active components of these mixtures were determined through re-synthesis and subsequent retesting. The screening data revealed a variety of heterocyclic urea derivatives, in particular ureas of 5-aminopyrazole (compounds **54**, Figure 8) and 2-aminothiazole, as inhibiting growth of gram-positive bacteria. In all cases, a disubstituted urea is necessary for activity and active compounds require, in addition to the pyrazole scaffold, an N-aryl substituent preferably 3,4 or 3,5 -disubstituted with halogen atoms and/or CF_3_ groups. However, despite the high in vitro activity (particularly against S. aureus compounds showed IC_50_ ranging from 0.054 to 4.24 μM), they were only marginally bioavailable under an in vivo test, probably due to their poor solubility.

### 4.9. 5-Pyrazolyl-Ureas as Anti-Trypanosome Agents

Improved therapies for the treatment of *Trypanosoma brucei*, the etiological agent of the neglected tropical disease human African trypanosomiasis, are urgently needed. *T. brucei* methionyl-tRNA synthetase (MetRS) is an aminoacyl-tRNA synthase (aaRS) that is considered an important drug target due to its role in protein synthesis, cell survival, and its significant differences in structure from its mammalian ortholog. 

In 2015, Pedro-Rosa and coworker [108] developed and applied to a large library of more than 300,000 compounds two orthogonal HTS assays to identify inhibitors of *T. brucei* MetRS. First, a chemiluminescence assay was implemented in a 1536-well plate format and used to monitor ATP depletion during the aminoacylation reaction. Hit confirmation then used a counter screen in which adenosine monophosphate production was assessed using fluorescence polarization technology. In addition, a miniaturized cell viability assay was used to triage cytotoxic compounds. Finally, lower throughput assays involving whole parasite growth inhibition of both human and parasite MetRS were used to analyze compound selectivity and efficacy. The outcome of this HTS campaign has led to the discovery of 19 potent and selective *T. brucei* MetRS inhibitors (having IC_50_ as low as 44 nM), including the 5-pyrazolyl-urea derivative **55** (Figure 8) (analogue of **51**, that belong to the series developed by Kortagere in 2010 [97]).

### 4.10. 5-Pyrazolyl-Ureas as Antiviral Agents

Respivert Limited UK in 2011 patented new compounds very similar to previous derivatives **24** and **25** (Figure 6), but bearing a more embedded imidazo[4,5-*b*]pyridine tail on the urea moiety. The most representative of this series is the 1-(3-(*tert*-butyl)-1-(4-methoxyphenyl)-1*H*-pyrazol-5-yl)-3-(4-((2-oxo-2,3-dihydro-1*H*-imidazo[4,5-*b*]pyridin-7yl)oxy)naphthalen-1-yl)urea **56** (Figure 8) claimed for the treatment of inflammatory diseases of respiratory system (asthma, COPD), and also for the treatment or prevention of inflammation mediated by viral infectious diseases (influenza virus, rhinovirus or RSV) [109]. All synthesized compounds were tested in anti-inflammatory, antiviral and cell viability assays. Moreover, compounds were tested in enzyme inhibition assays (p38MAPK, Src, Hck) in analogy to previous **24** and resulted active against p38MAPK with IC_50_s in the nanomolar range. An additional test on Rhinovirus-titration assay was performed, while results were not reported.

### 4.11. 5-Pyrazolyl-Ureas as Potassium Channel Activators

The G protein-gated inwardly-rectifying potassium channels (GIRK, K_ir_3) is a family of various homo- and hetero-tetrameric combinations of four different subunits expressed in different combinations in a variety of regions, throughout the central nervous system and in the periphery. A number of studies, using subunit-specific GIRK knockout mice, suggested the roles for GIRKs in a variety of important physiological processes and pointed to GIRK as a target for therapeutic intervention [110]. However, at the moment very few pharmacological tools have been reported that might allow a better understanding of the roles of GIRKs in normal and pathophysiological conditions. 

An HTS-compatible thallium flux assay for Gi/o-coupled GPCRs using GIRK as a readout [111] was used by Kaufmann and coworkers to screen the molecular libraries small molecule repository (MLSMR) as part of the Molecular Libraries Screening Center Network (MLSCN) [112]. The first efforts gave to the identification of the asymmetrical urea CID736191 (**57**, Figure 7) as activator of GIRK1/2 with an EC_50_ of ∼1 μM. This molecule was used as starting point for chemical optimization via iterative parallel synthesis. Several libraries were prepared surveying alternative substituents on the aryl ring, alternative heterocycles to replace the N-phenyl pyrazole, as well as alternative linkers for the urea moiety.

Testing of these compounds resulted in clear SAR. The N-phenyl pyrazole was essential for GIRK activity, as both NH moieties of the urea linker. In addition, 2-substituents to the aryl ring generally led to inactive compounds, whereas 3-substituted and 3,4-disubstitiuted analogues proved to be optimal. Chemical optimization afforded ML297 (VU0456810, CID 56642816, **58**, Figure 7), as the first potent GIRK activator. Since previous development of GIRK2 knockout animals has revealed an epilepsy phenotype, suggesting a role for GIRK in regulating excitability, and because the GIRK1/2 subunit combination is most prevalent in the brain, the authors reasoned that a GIRK1/2 activator might produce effects in an epilepsy model in vivo. Therefore, they evaluated ML297 in two model of epilepsy in mice (with PTZ or via electroshock). Despite the fact that ML297’s DMPK properties were suboptimal, robust activity was observed in both models. ML297 showed EC_50_ of 160 nM, equal or greater efficacy compared to a clinically active antiseizure medication, sodium valproate. These data support a role for selective GIRK activation in controlling excitability and provide the first evidence for the exciting possibility that GIRK may represent an attractive new target for antiepileptic drugs. 

Starting from this study, in 2017 the Wieting’s team reported a novel series of 1*H*-pyrazol-5-yl-2-phenylacetamides that showed nanomolar potency as GIRK1/2 activators with improved brain distribution in respect with the analogue urea derivatives [113].

## 5. Conclusions

In the last 20 years, linking of the pyrazole nucleus with a urea moiety has led to the design and synthesis of a lot of compounds with interesting biological activities. Many novel compounds have been synthesized and evaluated for their anti-pathogens (bacteria, plasmodium, toxoplasma and others), anti-inflammatory and, in particular, antitumor activity. The urea moiety has been identified as versatile scaffold for kinase inhibitors development, due to its unique binding mode and kinase inhibition profile. On the basis of X-ray crystallography studies, two distinct binding modes of urea binding to kinase have been elucidated: the urea function can bind with a conserved DFG residue (present in the activation loop of protein kinase) or with the hinge-binding site of protein kinase. In particular, urea moiety is used in the type II kinase inhibitors forming one or two hydrogen bonds with a conserved glutamic acid and another one with the backbone amide of the aspartic acid in the DFG motif. Moreover, this structure is used to link pharmacophores with high affinity DNA binder.

The most interesting results have been obtained when urea function was inserted in position 5 of the pyrazole scaffold, probably due to a simpler synthetic procedure. 5-Pyrazolyl-ureas have been largely investigated, being BIRB 796 the most studied compound as p38MAPK inhibitor, useful in autoimmune diseases treatment. 

As demonstrated by a multitude of patents reported in the literature from 2000, other important results have been obtained in anticancer field, where 5-pyrazolyl-ureas have shown to interact at intracellular level on many pathways, in detail on different kinases such as Src, Bcr-Abl, Flt3, TrkA and others. 

In addition, different 5-pyrazolyl ureas evidenced antiangiogenic and antiproliferative potential that deserves to be explored. 

Other important pharmacological results have been obtained in countering malaria, toxoplasma, trypanosome and bacterial infections, that, even today, owing the onset of multi-drug resistance, are not completely eradicated.

4-Pyrazolyl and 3-pyrazolyl ureas, although less investigated, showed varied pharmacological properties, from the inhibition of carbon anhydrase and epoxide hydrolase, to the action on potassium channels and on cannabinoid receptors. 

All these data confirm the importance of the urea function which is able to interact with different enzyme substrates depending on its different position on the pyrazole nucleus. Only in some cases, as widely reported above, both 4-pyrazolyl ureas and 5-pyrazolyl ureas have exhibited similar biological activity and have therefore been the subject of common patents.

For all these reasons, the design and synthesis of pyrazolyl-ureas are still ongoing and continue to give interesting insights into medicinal chemistry research.

This review summarizes the large number of pyrazolyl-ureas recently reported (from 2000 to date), including patented compounds and their related biological data.

## Figures and Tables

**Table 1:** Affinity constant and selectivity of CB1/CB2 antagonists **4a**–**d**.

	Affinity *K*_i_ (nM)		Selectivity
Comp. 4	CB1	CB2	CB1
**a**	9	4920	546.7
**b**	29	10863	374.6
**c**	6.8	4319	635.2
**d**	26	21791	838.1

## Abbreviations

3D-QSAR	Three-dimensional quantitative SAR
aaRS	Aminoacyl-tRNA synthase
Abl	Abelson kinase
AD	Alzheimer’s disease
ALS	Amyotrophic lateral sclerosis
AUDA	12-(1-Adamantan-1-ylureido)- dodecanoic acid
CAMK	Ca2+/calmodulin- dependent protein kinase
CB1/2	Cannabinoid receptors 1/2
CDK8/CycC	Cyclin-dependent kinase 8 and cyclin C
COMFA	Comparative molecular field analysis
CoMSIA	Comparative molecular similarity
COPD	Chronic obstructive pulmonary disease
COX-2	Cyclooxygenase-type 2
CXCL8	Interleukin 8
CXCR1/R2	CXC Chemokine receptors 1/2
DFG (DMG)	Asp-Phe-Gly motif
DMPK1	Dystrophia myotonica protein kinase 1
ERK	extracellular-regulated protein kinase
Flt3	Fms related tyrosine kinase 3
fMLP	N-formyl-methionyl-leucyl-phenylalanine
GIRK	G protein-gated inwardly-rectifying potassium channels
GSK3α	Glycogen synthase kinases type 3α
hCA II	Human carbonic anhydrase II
hCAIs	Human carbonic anhydrase inhibitors
Hck	Hemopoietic Cell Kinase
HD	Huntington’s disease
HeLa	Henrietta Lacks immortal cells
hERG	Human Ether-à-go-go-Related Gene
HFF	Human foreskin fibroblasts
HSB	Hybrid structure based (virtual screening approach)
HTS	High-throughput screening
HUVEC	Human umbilical vein endothelial cells
I-κB	Inhibitor of kappa B kinase
IKK (1/2)	IkB kinases type 1/2
IL-1-/6/-17/-18	Interleukin type 1/6/17/18
JM	Juxta-membrane
JNK	c-Jun NH_2_-terminal kinase
LPS	Lipopolysaccharide
MAPK	Mitogen-activated protein kinase
MBC	Minimum Bactericidal concentration
MES	Maximal electroshock seizure
MetRS	Methionyl-*t*-RNA synthetase
MFC	Minimum fungicidal concentration
MI	Myocardial infarction
MIC	Minimum inhibiting concentration
MLSCN	Molecular libraries screening center network
MLSMR	Molecular libraries small molecule repository
MS	Multiple sclerosis
MTIP	Myosin tail interacting protein
MyoA	Myosin A
NEK10	NIMA (never in mitosis gene a)-related kinase 10
NF-κΒ	Nuclear factor kappa-light-chain-enhancer of activated B cells
NGF	Nerve growth factor
ONIOM	N-Layered integrated molecular orbital & molecular mechanics
Panc-1	Human pancreatic adenocarcinoma
PD	Parkinson’s disease
PDB	Protein data bank
PDGFR	Platelet derived growth factor
PfATP4	Plasmodium falciparum p-type cation ATPase
PfProRS	Plasmodium falciparum prolyl-*t*-RNA-synthetase
PI3K	Protein inositol kinase 3
PKC	Protein kinase C
PLK2, PLK3	Polo-like kinase 2/3
PPI	Protein-protein interaction
PTZ	Pentylenetetrazol-induced seizure
RAF/RAS	Rapidly accelerated Fibrosarcoma
RSV	Respiratory syncytial virus
SAR	Structure Activity Relationship
sEH	Soluble epoxide hydrolases
SUMO	Small ubiquitin-like modifier
Syk	Spleen tyrosine kinase
TNF-α	Tumor necrosis factor alpha
Trk	Tropomyosin receptor kinase
UBSAs	Ureido benzenesulfonamides
VEGF	Vascular endothelial growth factor
VEGFR1/2	Vascular endothelial growth factor receptor
Zap-70	Zeta Chain of T Cell Receptor Associated Protein Kinase 70
